# Fibronectin-targeting and metalloproteinase-activatable smart imaging probe for fluorescence imaging and image-guided surgery of breast cancer

**DOI:** 10.1186/s12951-023-01868-5

**Published:** 2023-03-28

**Authors:** Zhongquan Cheng, Yushen Jin, Jiaqian Li, Guangyuan Shi, Leyi Yu, Bing Shao, Jie Tian, Yang Du, Zhu Yuan

**Affiliations:** 1 Department of General Surgery, Capital Medical University, Beijing Friendship Hospital, Beijing, 100050 China; 2 CAS Key Laboratory of Molecular Imaging, Beijing Key Laboratory of Molecular Imaging, Institute of Automation, Chinese Academy of Sciences, Beijing, 100190 China; 3 Beijing Key Laboratory of Diagnostic and Traceability Technologies for Food Poisoning, Beijing Center for Disease Prevention and Control, Beijing, 100013 China; 4 School of Biological Science and Medical Engineering, Beihang University, Beijing, 100083 China; 5 University of Science and Technology of China, Hefei, 230026 Anhui China; 6 Haidian Section of Peking University Third Hospital, Beijing, 100080 China; 7 University of Chinese Academy of Sciences, Beijing, 100080 China; 8 Beijing Advanced Innovation Center for Big Data-Based Precision Medicine, School of Medicine Science and Engineering, Beihang University, Beijing, 100191 China

**Keywords:** Matrix metalloproteinase-9, Fibronectin, Fluorescence molecular imaging, Image-guided surgery, Breast cancer

## Abstract

Residual lesions in the tumor bed have been a challenge for conventional white-light breast-conserving surgery. Meanwhile, lung micro-metastasis also requires improved detection methods. Intraoperative accurate identification and elimination of microscopic cancer can improve surgery prognosis. In this study, a smart fibronectin-targeting and metalloproteinase-activatable imaging probe CREKA-GK8-QC is developed. CREKA-GK8-QC possesses an average diameter of 21.7 ± 2.5 nm, excellent MMP-9 protein responsiveness and no obvious cytotoxicity. In vivo experiments demonstrate that NIR-I fluorescence imaging of CREKA-GK8-QC precisely detects orthotopic breast cancer and micro-metastatic lesions (nearly 1 mm) of lungs with excellent imaging contrast ratio and spatial resolution. More notably, fluorescence image-guided surgery facilitates complete resection and avoids residual lesions in the tumor bed, improving survival outcomes. We envision that our newly developed imaging probe shows superior capacity for specific and sensitive targeted imaging, as well as providing guidance for accurate surgical resection of breast cancer.

**Graphical Abstract:**

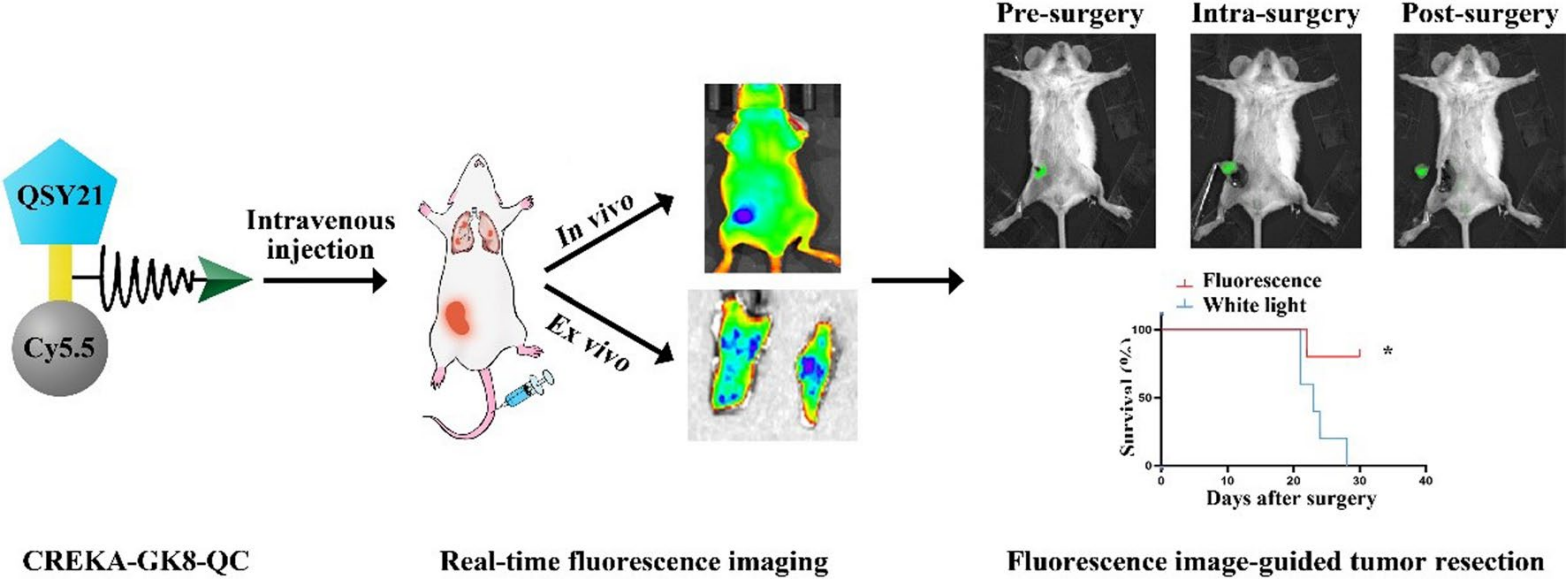

**Supplementary Information:**

The online version contains supplementary material available at 10.1186/s12951-023-01868-5.

## Introduction

In 2020, breast cancer was the most women diagnosed cancer [1]. Currently, surgery is an important tool for cancer eradication [2, 3]. During the surgical process, most surgeons rely heavily on their eye and experience to evaluate the in vivo lesions. However, these subjective factors are unreliable leading to residual lesions [4]. One study demonstrated that ipsilateral breast cancer recurrence could be detected in 4.1% of patients assigned to only breast conserving surgery (BCS) within 5 years of follow-up [5]. Therefore, conventional white light breast surgery failed to eliminate all malignant cells [6, 7]. Additionally, lung metastases are often a cluster of cancer cells less than 5 mm in diameter, which are generally diagnosed using preoperative imaging [8]. Owing to the current low detection rate (less than 20%) and limited accessibility of intraoperative diagnostic methods, it is urgent to develop novel imaging tools that can be applied for precise identification of primary breast tumors, lung metastases, and further image-guided surgery.

For years, molecular imaging (MI) has been applied to image microscopic lesions of cancer [9, 10]. Based on novel imaging probes, Fluorescence molecular imaging (FMI) can differentiate malignant lesions from benign tissues with obvious contrast ratios [11, 12]. Nowadays, FMI has been reported for ex vivo specimen evaluation, in vivo tumor-targeted imaging, and other clinical uses [13–16], which facilitated the accurate detection and treatment of breast cancer.

As important biomarkers, matrix metalloproteinase-9 (MMP-9) correlates closely with cancer invasion and is overexpressed within breast cancer tissues, especially in triple-negative breast cancer (TNBC) [17–19]. Targeting to MMP-9, one study has achieved highly sensitive imaging of cancer [20]. However, MMP-9 is also expressed at a relatively higher level in inflammatory disease than normal breast tissues. Only MMP-9-targeting strategy may impact the specificity of FMI [21]. Simultaneously, fibrin-fibronectin complexes within extracellular microenvironment stood for vital imaging biomarkers for specific delivery of imaging probes to the cancerous site [22]. Fibronectin-targeting strategies have been applied for detection and image-guided treatment of breast cancer [16, 23, 24]. Hence, it is meaningful to synthesize a dual-targeted imaging probe that combines MMP-9- and fibronectin-targeting abilities for accurate diagnosis and FMI-guided surgery for breast cancer.

**Fig. 1:**
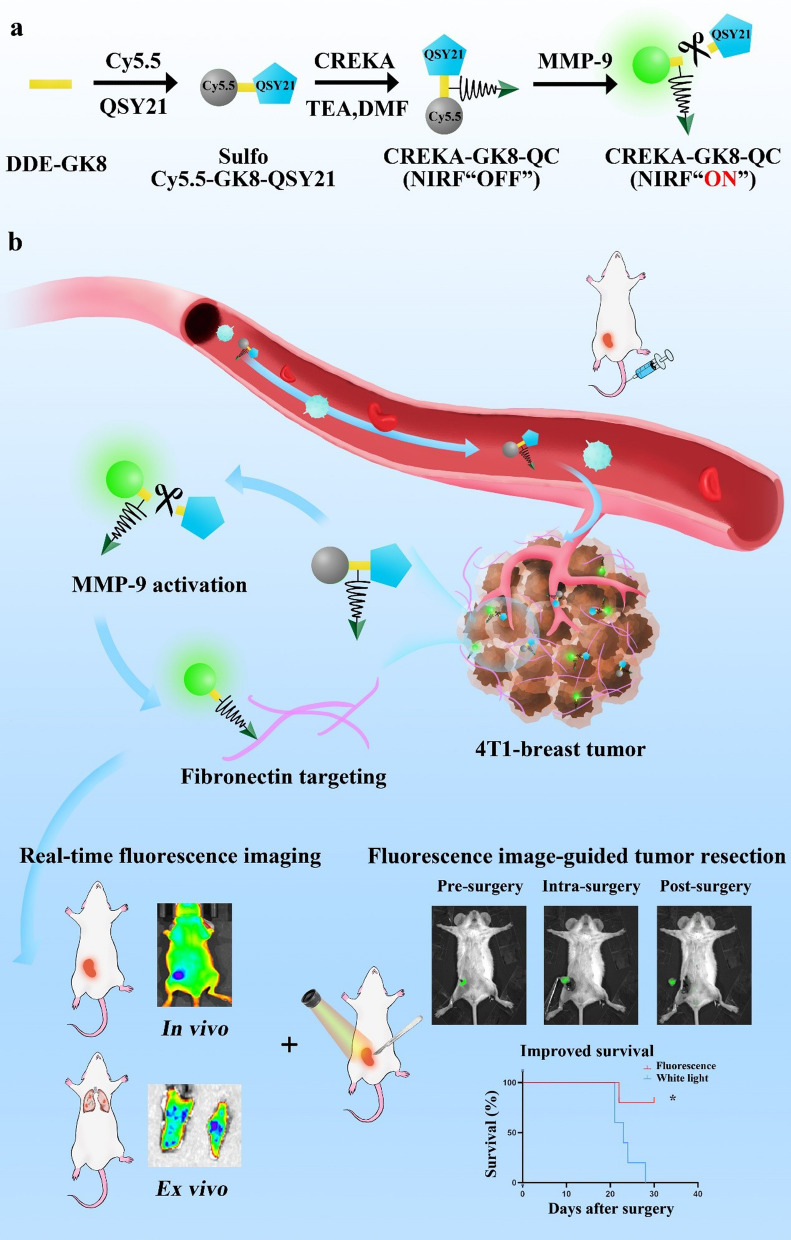
Schematic illustration of the synthesis and workflow of the CREKA-GK8-QC imaging probe. **a**. MMP-9-cleavable peptide sequence GPVGLIGK was applied to covalently link a quencher (QSY21), a NIR dye (Cy5.5), and a fibronectin-targeting peptide CREKA to synthesize dual-targeted fluorescence imaging probe CREKA-GK8-QC. This probe in the intact state is optically silent at the beginning, but upon proteolytic cleavage with MMP-9 enzyme, it results in separation of the QSY21/Cy5.5 pair and loss of fluorescence resonance energy transfer to emit a strong NIR fluorescence signal. **b**. After intravenous injection, CREKA-GK8-QC could target to the tumor sites through CREKA targeting, and the fluorescence signal could be detected upon the cleavage on the peptide sequence GPVGLIGK by extracellular MMP-9 protein This MMP-9-activable fibronectin-targeted imaging probe, CREKA-GK8-QC, could facilitate the specific detection of in vivo breast cancer, ex vivo lung metastasis, and further precise fluorescence image-guided resection of breast cancer

In this study, we developed a novel strategy that targeted both MMP-9 and fibronectin in the tumor microenvironment (TME). As depicted in Fig. 1a, the MMP-9-cleavable peptide sequence GPVGLIGK (GK8) [25] was used to covalently link the NIR dye Cy5.5, its quencher QSY21, and the fibronectin-targeting peptide Cys-Arg-Glu-Lys-Ala (CREKA) [26] to construct a dual-targeted near infrared-I (NIR-I) imaging probe, QSY21-GPVGLIGK-CREKA-Cy5.5, which was named as CREKA-GK8-QC. The CREKA-GK8-QC molecules can form uniform nanoparticles in aqueous solution through spontaneous self-assembly because there were significant differences in hydrophilicity and lipophilicity among QSY21, Cy5.5, and CREKA molecules. CREKA-GK8-QC could successfully bind to the overexpressed fibronectin and its fluorescence could be specifically activated by MMP-9 protein in the TME. These characteristics make CREKA-GK8-QC a promising tool for the precise diagnosis of primary lesions and lung micro-metastasis and image-guided surgery (Fig. 1b).

## Experimental section

### Materials

Sulfo Cy5.5 succinimidyl ester (sulfo Cy5.5-NHS) was purchased from Sigma-Aldrich (Shanghai China). MMP-9-activable peptide (DDE-protected peptide GPVGLIGK, DDE-GK8), fibronectin-targeted peptide (CREKA), and non-target peptide (CERAK) were synthesized by Qiangyao Biotech (Suzhou, China). QSY21 carboxylic acid succinimidyl ester (QSY21-NHS) was obtained from Molecular Probes (Eugene, OR). O-(6-Chlorobenzotriazol-1-yl)-N,N,N′,N′,- tetramethyluronium tetrafluoroborate (TCTU) was purchased from Adamas Reagent Co., Ltd. (Shanghai, China). 1-Ethyl-(3-dimethylaminopropyl) carbondiimide hydrochloride (EDC), N-hydroxysuccinimide (NHS), dimethyl sulfoxide, trifluoroacetic acid, trimethylamine (TEA), and other chemicals were obtained from Sigma-Aldrich (Shanghai, China). MMP-9 was obtained from R&D Systems, Inc. (Minneapolis, MN, USA). The MMP-9 inhibitor GM6001 was purchased from TargetMol Chemicals, Inc. (Boston, MA, USA).

### Synthesis and characterization of CREKA-GK8-QC

The synthesis of CREKA-GK8-QC involved three steps. First, QSY21-NHS and DDE-GK8 at a molar mass ratio of 1:1.2 were dissolved in 5 mL of dimethylformamide (DMF). Then, 20 μL TEA was added to the mixture, and the reaction was carried out under nitrogen protection for approximately 24 h at 40 °C. The synthesized QSY21-GK8-DDE was purified by dialysis against deionized water. Then, the purified QSY21-GK8-DDE was added to 2% hydrazine hydrate dissolved in dichloromethane (DCM) and incubated for 30 min to remove DDE protection and expose the amino group. Finally, DCM was removed by rotary evaporation.

QSY21-GK8 and Cy5.5-NHS at a molar mass ratio of 1:1.2 were dissolved in 5 mL DMF containing 5% TCTU and 20 μL TEA, and the reactions were also carried out under the nitrogen protection for approximately 24 h at room temperature to obtain QSY21-GK8-CY5.5. The synthesized QSY21-GK8-CY5.5 was also purified by dialysis against deionized water.

**Fig. 2:**
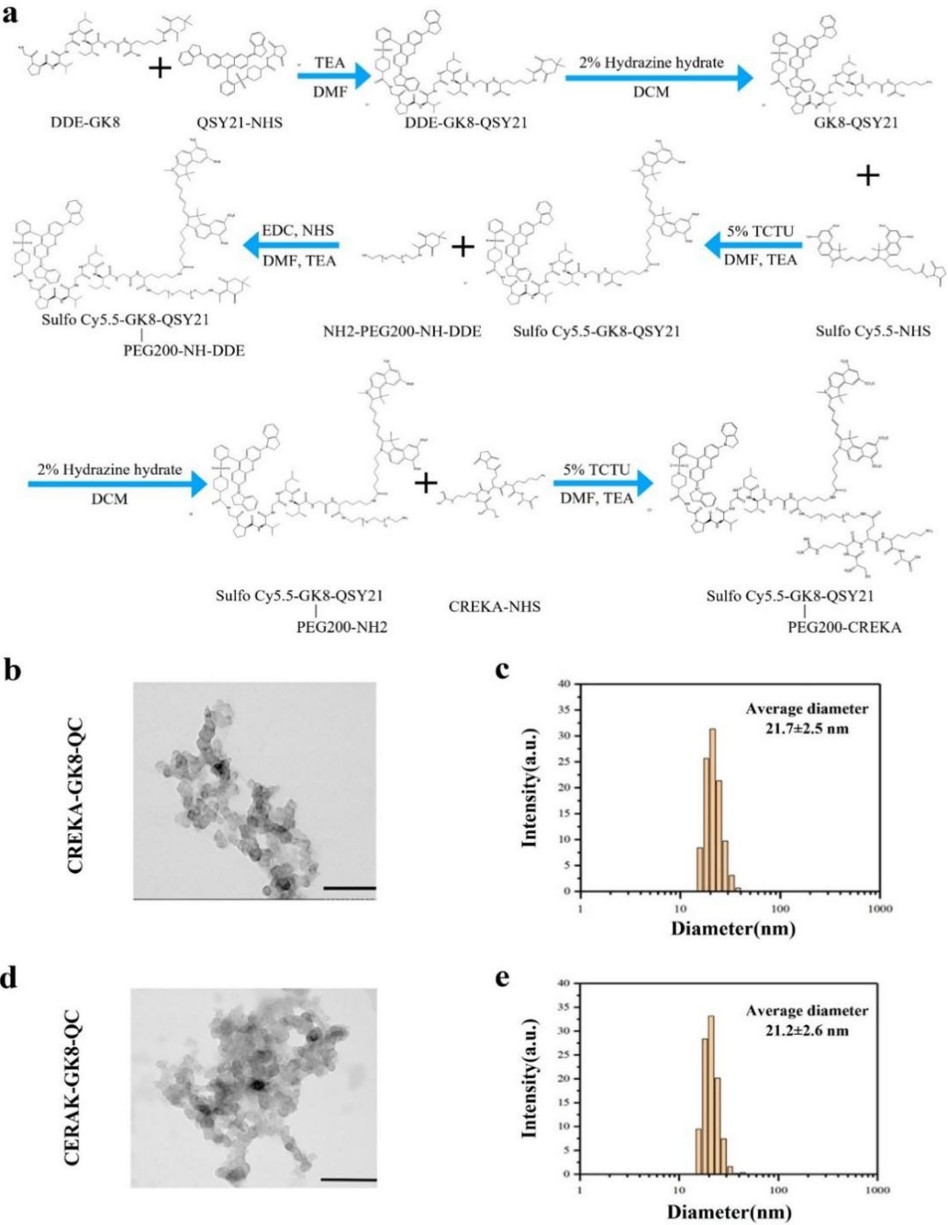
Characterization of the CREKA-GK8-QC imaging probe. **a**. Synthesis route of CREKA-GK8-QC. **b**. Transmission electron microscopy images of targeted CREKA-GK8-QC (scale bar: 200 nm). **c**. Size analysis of targeted CREKA-GK8-QC by transmission electron microscopy. **d**. Transmission electron microscopy images of control CERAK-GK8-QC (scale bar: 200 nm). **e**. Size analysis of control CERAK-GK8-QC by transmission electron microscopy

Purified QSY21-GK8-CY5.5 was dissolved in 5 mL DMF containing EDC and NHS, and the mixture was stirred for 30 min in an ice bath. Then, 20 μL of TEA and NH_2_-PEG200-NH-DDE were added to the mixture, and the reaction was carried out under nitrogen protection for 24 h at room temperature. The DDE protection was removed using a method similar to that described above. Then, PEG-modified QSY21-GK8-CY5.5 was reacted with CREKA peptide in 5 mL DMF containing EDC, NHS, and 20 μL triethylamine under nitrogen protection for 24 h at room temperature. Impurities and unreacted raw materials were removed by dialysis against deionized water to purify the obtained CREKA-GK8-QC. Detailed information on the synthesis route of CREKA-GK8-QC is shown in Fig. 2a. The control CERAK-GK8-QC was synthesized in a similar manner.

During synthesis, sulfo Cy5.5-GK8-QSY21, CREKA-GK8-QC, and CERAK-GK8-QC were lyophilized and characterized by high-resolution mass spectrometry (HR-MS, Xevo G2-XS QTof Quadrupole Time-of-Flight Mass Spectrometry). The morphology and size distribution of CREKA-GK8-QC and CERAK-GK8-QC in deionized water were measured using a JEOL JSM-100SX instrument (JEOL, Japan) operating at 100 kV and dynamic light scattering (DLS, Malvern Instruments, England), respectively.

### Enzymatic assays

In vitro enzymatic analysis was performed in 200 μL buffer solution (20 mM HEPES, pH 7.4). A variety of different concentrations of recombinant MMP-9 enzyme (0, 25, 50, 100, 200, 400 ng/mL) were incubated with CREKA-GK8-QC (5 μM) for 2 h at 37 °C in 96-well black plates, respectively. For competitive analysis, the MMP-9 inhibitor (GM6001, 150 μM) was first incubated with MMP-9 (400 ng/mL) for 1 h, followed by the addition of 5 μM CREKA-GK8-QC incubated for another 2 h at 37 °C. Subsequently, the mixture was imaged using an IVIS Lumina System (PerkinElmer). The same MMP-9-responsive and -competitive analysis of the contrast imaging probe CERAK-GK8-QC was also performed.

### Cell culture

The murine breast carcinoma cell line 4T1-fLuc was obtained from American Tissue Culture Center (Manassas, VA, USA). The 4T1-fLuc cells were maintained in RPMI 1640 medium (Hyclone Inc.) supplemented with 10% fetal bovine serum (Hyclone Inc.) and 1% penicillin streptomycin (Beyotime Inc.) at 37 °C in a humidified atmosphere of 5% CO_2_. When the cells reached 80–90% confluence, the cells were subcultured for the further experiments.

### Western blot

The 4T1 tumors and normal mammary glands of the mice were collected and homogenized in 500 μL cold T-PER buffer (Thermo Fisher Scientific) containing a protease inhibitor cocktail (Sigma-Aldrich). Then, centrifugation at 10,000 × g for 15 min at 4 °C was applied to remove insoluble components. Protein concentrations were analyzed by BCA assay (Beijing ComWin Biotech). The samples were incubated with anti-MMP-9 polyclonal antibody (Abcam), anti-fibronectin polyclonal antibody (Abcam), or anti-GAPDH antibody (Abcam) at 4 °C overnight, respectively. After washing 3 times in TBST, the above supernatants were incubated with the secondary antibody horse-radish peroxidase (HRP)-conjugated goat anti-rabbit IgG for 1 h at 24 °C. ECL reagents were then added, and western blot images were captured.

### In vitro* cytotoxicity analysis*

The Cell Counting Kit-8 (CCK-8, Solarbio) was used for *the *in vitro cytotoxicity analysis. 4T1 cells were seeded in a 96-well plate at a density of 5000 cells per well in 200 µL RPMI 1640 medium. Each group include five parallel wells. After incubation for 24 h, various concentrations of CREKA-GK8-QC were added to the substrates and incubated for another 24 h. Cells in culture medium were used as controls. Finally, CCK-8 reagent was added, and a VICTOR Nivo Reader (PerkinElmer) was used to detect the absorbance of each well at 450 nm.

### Animal tumor models

6-week-old female BALB/c and athymic nude mice were purchased from Beijing Vital River Laboratory Animal Technology Co., Ltd. All animal experiments were performed according to a protocol approved by the Institution of Automation, Chinese Academy of Sciences (Permit No: IA21-2203-24). 4T1-fLuc cells (5 × 10^5^ cells in 50 μL PBS) were injected into the right mammary fat pad, lower right thigh, or tail vein of the mouse to establish orthotopic breast tumors, subcutaneous breast tumors, or breast cancer with lung metastatic lesions, respectively.

### In vivo* fluorescence imaging (FMI) analysis*

The orthotopic breast tumor, subcutaneous breast tumor, and lung metastasis mouse models were intravenously injected with the targeted CREKA-GK8-QC or control CERAK-GK8-QC imaging probe at a dose of 100 µg/mL (n = 3). IVIS Imaging Spectrum System (PerkinElmer Inc.) was used to perform FMI after all mice were anesthetized with 2% isoflurane. Data were dynamically obtained from 0 to 48 h after intravenous administration of the imaging probe and analyzed using the IVIS Living Imaging software (version 4.0; PerkinElmer Inc.). To evaluate the biodistribution and tumor-targeting effects, the fluorescence signal was collected and reported as the average radiant efficiency (p/sec/cm^2^/sr), and the tumor-to-background ratio (TBR) was also calculated. Then *ex vivo* FMI analysis was performed 48 h after *in vivo* FMI.

### Fluorescence image-guided breast surgery

Ten 4T1-fLuc orthotopic tumor-bearing mice were randomly divided into two groups: fluorescence image-guided surgery and conventional white light surgery (n = 5). After the tumor volume reached ~ 100 mm^3^, all mice were intravenously injected with CREKA-GK8-QC, and surgery was performed 8 h later. For the fluorescence image-guided surgery group, real-time intraoperative fluorescence image-guided tumor resection was performed using a fluorescence stereomicroscope (M205 FA; Leica Microsystems, Wetzlar, Germany) with the NIRF setting (excitation:675 nm, emission:690 nm). For conventional white light surgery, surgeons perform tumor resection according to their perception and experience, no aid of intraoperative fluorescence guidance. Bioluminescent imaging (BLI, Caliper Life Sciences, USA) was also performed pre- and postoperatively to detect residual tumor.

### Analysis of post-surgical tumor recurrence

Tumor recurrence (through BLI) and body weight of the mice were monitored every 3 days for 30 days post-surgery. The survival rate of mice was also monitored during the 30 day observation.

### Histological analysis

After in vivo fluorescence imaging, the breast tumor tissues were dissected and preserved in 4% paraformaldehyde for further embedding and sectioning. All breast tumor samples were sectioned at a thickness of 4 μm for further observation. Hematoxylin and eosin (H&E) staining was used to indicate histological changes and scanned using a PANNORAMIC MIDI II scanner (3DHISTECH, Hungary). For immunohistochemistry, tumor tissues were stained according to standard protocols. Rabbit anti-mouse MMP-9 polyclonal antibody (Abcam, UK) and rabbit anti-mouse fibronectin polyclonal antibody (Abcam, UK) were used for staining. For immunofluorescence staining, the above two polyclonal antibodies were applied to stain MMP-9 and fibronectin, followed by FITC-labeled goat anti-rabbit IgG. Finally, the nuclei were stained with DAPI solution for 10 min at 24 °C in the dark. Immunofluorescence images were obtained using a Leica SP8 STED 3X system.

### Statistical analysis

GraphPad Prism software (Version 8.0) was used for statistical analysis. Data are presented as the mean ± standard deviations (SD). Statistical assessment was carried out by Student’s t test for two groups or one-way ANOVA within multiple groups. Overall survival was evaluated by using Kaplan–Meier analysis. *p < 0.05 was considered as statistical significance.

## Results

### Characterization of the MMP-9 and fibronectin expression

To validate whether MMP-9 and fibronectin could serve as specific imaging targets for breast cancer, their expression was analyzed in orthotopic 4T1 breast tissue and normal breast tissue. As shown in Additional file 1: Figs. S1, S2, we found that higher MMP-9 and fibronectin expression levels were detected in orthotopic breast tumors than in normal breast tissues. Similarly, these results were also observed in the lung metastases compared to normal lungs (Additional file 1: Figs. S3, S4). Furthermore, we analyzed the expression levels of MMP-9 and fibronectin through western blot in the breast tumors and normal breast tissues of orthotopic 4T1 breast tumor-bearing mice. Western blot analysis clearly indicated that MMP-9 and fibronectin were both overexpressed in the breast tumor tissues (Additional file 1: Figs. S5, S6). Statistical analysis demonstrated that the expression levels of MMP-9 in breast tumors were higher (~ 7.33-fold, **p < 0.01) than those in normal breast tissues (Additional file 1: Fig. S5). The expression level of fibronectin was also higher in breast tumors (~ 10.59-fold, **p < 0.01) than in normal tumors (Additional file 1: Fig. S6). These results suggested that both MMP-9 and fibronectin were highly expressed in breast tumors and could serve as specific imaging biomarkers for further fluorescence imaging and FMI-guided surgery.

### Characterization of CREKA-GK8-QC imaging probe

The targeted probe (CREKA-GK8-QC) and control probe (CERAK-GK8-QC) were synthesized using a method similar to that reported by Fang et al. [25]. Briefly, the MMP-9-responsive peptide sequence DDE-GK8 was first conjugated with Cy5.5 (Cy5.5-NHS) in 5 mL DMF containing 20μL TEA, and after removal of the DDE protection, reacted with QSY21-NHS, followed by purification through dialysis against deionized water to obtain Cy5.5-GK8-QSY21, which is denoted as GK8-QC below. Finally, CREKA-NHS or CERAK-NHS was conjugated to GK8-QC through a functionalized PEG linker to obtain the desired targeted and control probe, respectively (Fig. 2a).

The purity of imaging probes was characterized using HR-MS (Additional file 1: Figs. S7, S8, S9). Calcd. for sulfo Cy5.5-GK8-QSY21, ([M-3 K + 2H]^2+^), ([M-3 K + 3H]^3+^) and ([M-3 K + 4H]^4+^): 1160.8943, 774.2655 and 580.9511, respectively, found ESI–MS: m/z 1160.8902, 774.2635 and 580.9471, respectively; Calcd. for CREKA-GK8-QC and control CERAK-GK8-QC; ([M-3 K + 3H]^3+^) and ([M-3 K + 4H]^4+^): 1056.0374 and 792.2778, respectively; ESI–MS: m/z 1056.0354 and 792.2756, respectively. The CREKA-GK8-QC and control CERAK-GK8-QC molecules formed uniform nanoparticles in an aqueous solution through spontaneous self-assembly, as shown in Fig. 2b, d. The average diameters of the CREKA-GK8-QC and control CERAK-GK8-QC were 21.7 ± 2.5 nm and 21.2 ± 2.6 nm, respectively (Fig. 2c, e).

### In vitro* MMP-9 responsiveness, targeting specificity and cytotoxicity of CREKA-GK8-QC*

**Fig. 3:**
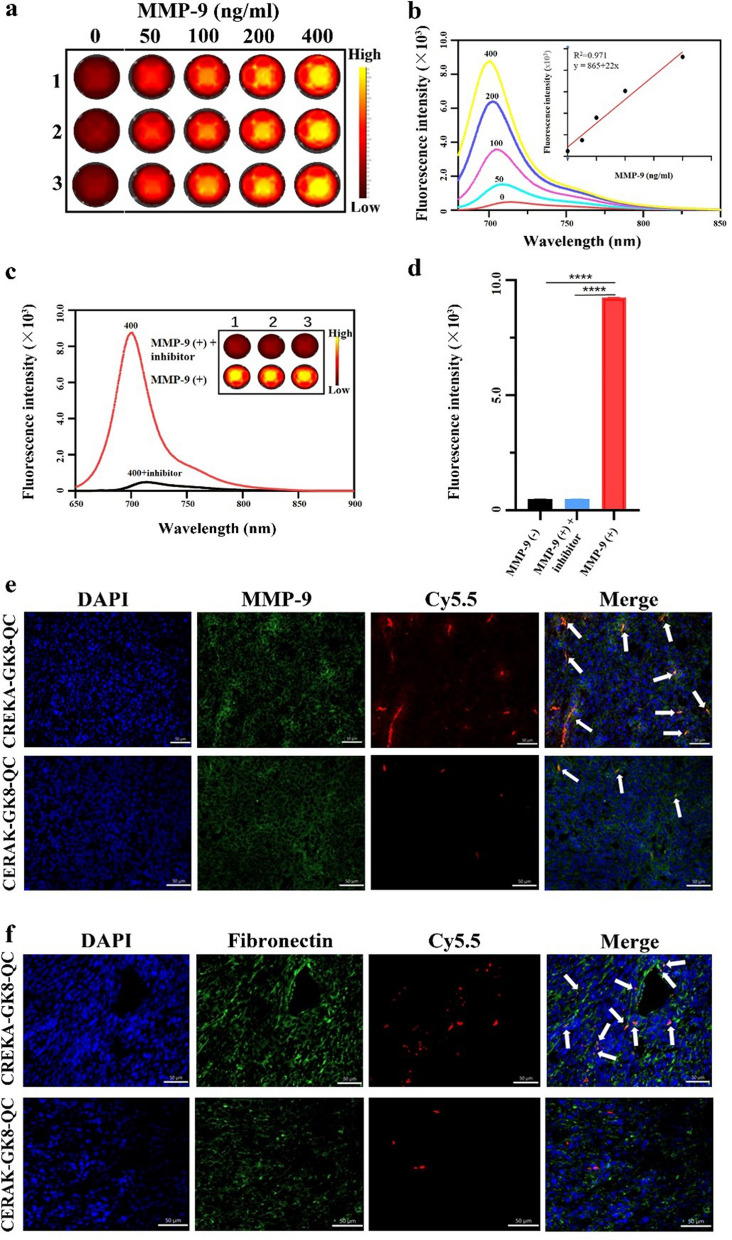
In vitro MMP-9 responsiveness, targeting specificity of CREKA-GK8-QC. **a**. In vitro fluorescence images of CREKA-GK8-QC (5 μM) treated with 0, 50, 100, 200, and 400 ng/mL of MMP-9 protein at 37 °C for 2 h using IVIS spectrum. **b**. The fluorescence spectra of CREKA-GK8-QC after incubation with various concentrations of MMP-9 (0, 50, 100, 200, and 400 ng/mL) (inset: MMP-9 concentration-dependent fluorescence intensity of CREKA-GK8-QC). **c**. The fluorescence spectra results of CREKA-GK8-QC after incubation with MMP-9 (400 ng/mL), a mixture of MMP-9 and its inhibitor GM6001 (150 μM) (inset: the fluorescence images of the above solutions were recorded on an IVIS system). **d**. Fluorescence intensity of CREKA-GK8-QC treated with 0 ng/mL, 400 ng/mL, and combined 150 μM MMP-9 inhibitor and 400 ng/mL of MMP-9 protein using the ultraviolet–visible spectrophotometer, ****p < 0.0001. **e**. Analysis of MMP-9 expression and distribution of targeted CREKA-GK8-Cy5.5 and control CERAK-GK8-QC in 4T1 breast tumor sections, respectively. **f**. Analysis of fibronectin expression and binding of targeted CREKA-GK8-QC and control CERAK-GK8-QC to 4T1 breast tumor sections, respectively. DAPI was used for nuclei staining. Pseudo-colors in the confocal images are assigned as follows: red, Cy5.5; green, fibronectin; and blue, nucleus. Scale bar 50 µm

As shown in Fig. 3a, CREKA-GK8-QC exhibited an excellent capacity to respond to MMP-9, and the fluorescence intensity gradually increased and correlated well with the concentrations of MMP-9 from 0 to 400 ng/mL. When the concentration of MMP-9 was lower than 400 ng/mL, the fluorescence intensity increased linearly with the MMP-9 concentration (R^2^ = 0.97), which demonstrated that CREKA-GK8-QC manifested a significant increase in fluorescence signal intensity with increasing MMP-9 concentrations (Fig. 3b). Otherwise, activation could be effectively blocked by pretreatment of CREKA-GK8-QC with GM6001 (an inhibitor of MMP-9 proteins) (Fig. 3c). The average fluorescence intensity of the MMP-9-treated group was significantly higher than that of the non-MMP-9-treated and GM6001-treated group (****p < 0.0001) (Fig. 3d). These results demonstrated that CREKA-GK8-QC exhibited excellent responsiveness to MMP-9 in vitro. The specificity of CREKA-GK8-QC binding to MMP-9 and fibronectin in the 4T1-tumor-bearing mice was verified through immunofluorescence staining. 4T1 orthotopic tumor sections were rich in MMP-9 and fibronectin expression, and after treatment with CREKA-GK8-QC, strong Cy5.5 fluorescence was observed. However, little to almost no Cy5.5 fluorescence was detected in the control CERAK-GK8-QC group (Fig. 3e, f). Subcutaneous breast tumors and metastatic lesions showed similar results (Additional file 1: Figs. S10, S11). Taken together, these results demonstrated the specific binding of CREKA-GK8-QC to MMP-9 and fibronectin in breast tumor tissues. At the same time, as shown in Additional file 1: Fig. S12, the cytotoxicity assay showed that CREKA-GK8-QC exhibited negligible cytotoxicity on 4T1 cells in the concentration range of 0–200 μg/mL. The overall cell viability was > 75%.

### In vivo* NIR fluorescence imaging of orthotopic and subcutaneous breast cancer through CREKA-GK8-QC*

**Fig. 4:**
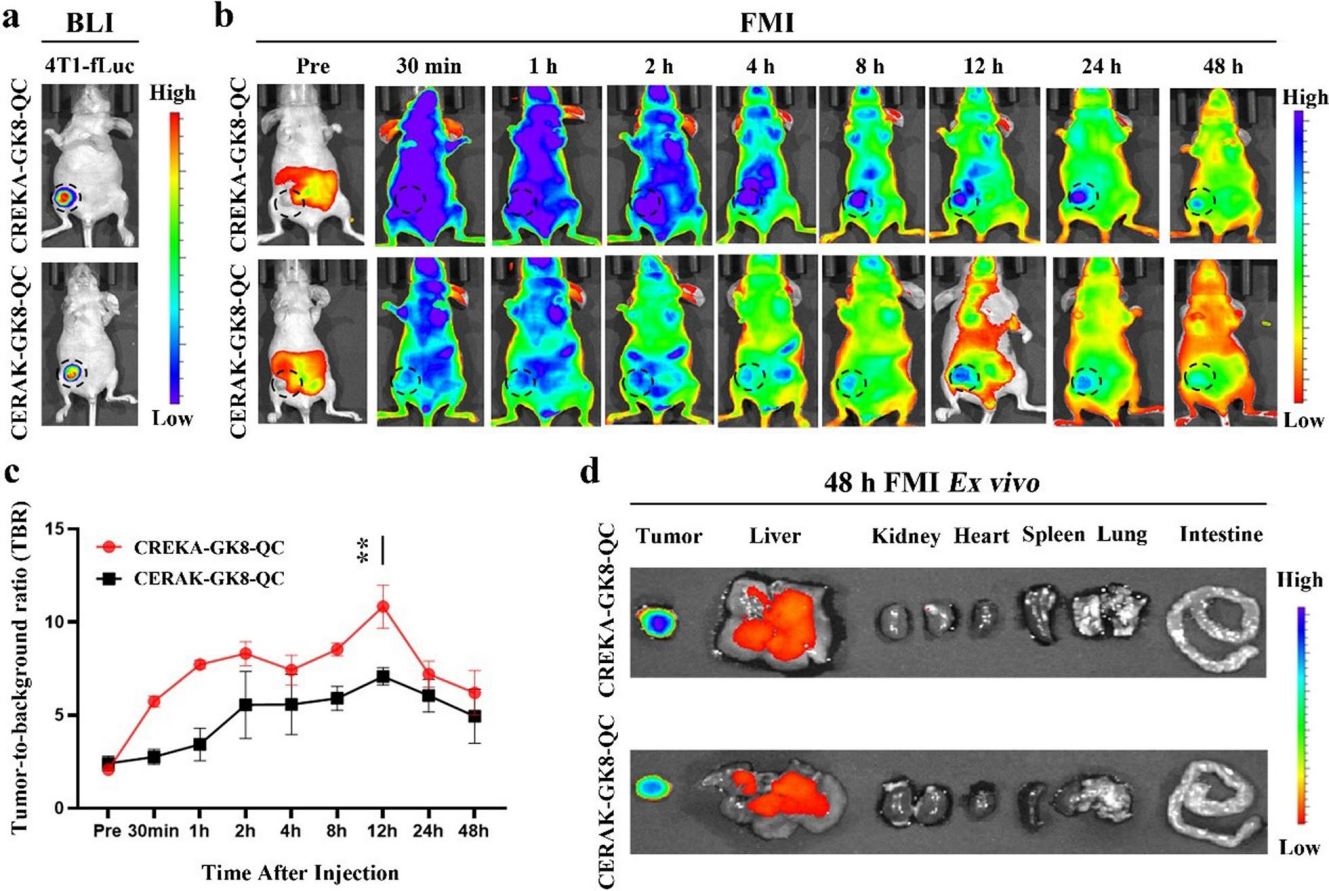
In vivo fluorescence imaging of CREKA-GK8-QC in orthotopic 4T1 breast cancer model. **a**. BLI of orthotopic 4T1 breast tumor-bearing mice was used to detect the location of the tumors. **b**. In vivo fluorescence images of orthotopic breast tumor-bearing mice injected with CREKA-GK8-QC or CERAK-GK8-QC (n = 3) at different time points. The dotted black circles indicate the locations of orthotopic breast tumors. **c**. Quantification of in vivo fluorescence imaging of tumor to background ratio (TBR). Data are represented as the mean ± SD, n = 3, **p < 0.01. **d**. Ex vivo fluorescence imaging of tumors and major organs, including liver, kidney, heart, spleen, lung, and intestine, at 48 h post-injection of CREKA-GK8-QC or CERAK-GK8-QC

Based on these findings, the in vivo tumor-targeting abilities and biodistribution of CREKA-GK8-QC were analyzed in an orthotopic 4T1 breast tumor mouse model using NIR fluorescence imaging after the tumor volume reached approximately 100 mm^3^. BLI was used to detect the location of the orthotopic breast tumor (Fig. 4a), confirming the successful establishment of an orthotopic breast tumor. NIR fluorescence imaging was performed at different time points after injection of CREKA-GK8-QC. As shown in Fig. 4b, FMI results indicated that CREKA-GK8-QC localized to the orthotopic breast tumor 30 min post-injection, gradually accumulated from 1 to 12 h, and then decreased thereafter with a weak signal detected at 48 h. For the CERAK-GK8-QC control group, nonspecific fluorescence signals were also identified in the tumor region from 1 to 12 h post-injection, and the tumor-localized signal decreased until 48 h. Quantification of the TBR for each group demonstrated that the CREKA-GK8-QC signal was more specific and stable within the tumor region than the CERAK-GK8-QC control group. The highest TBR was detected at 12 h, which was approximately 1.53-fold higher than that in the CERAK-GK8-QC control group (**p < 0.01) (Fig. 4c). Furthermore, orthotopic breast tumors and major organs were collected at 48 h post-injection (Fig. 4d), and their fluorescence signals were quantitatively calculated (Additional file 1: Fig. S13). Tumors possessed the highest fluorescence signals compared with other organs and the fluorescence signals of breast tumors in the CREKA-GK8-QC group were obviously stronger than those in the CERAK-GK8-QC group (21.33 ± 2.62 vs. 15.33 ± 1.36, respectively, *p < 0.05). Collectively, these results indicated that CREKA-GK8-QC exhibited outstanding tumor-targeting abilities and possessed the potential for intraoperative image guidance for the surgical treatment of orthotopic breast tumors.

Meanwhile, the in vivo tumor-targeting effects and biodistribution of CREKA-GK8-QC were also evaluated in a subcutaneous 4T1 breast tumor mouse model at different time points after intravenous injection of 100 µL CREKA-GK8-QC. Similar FMI results were observed and the CREKA-GK8-QC signal was more specific and stable within the tumor region than the CERAK-GK8-QC control group (Additional file 1: Figs. S14, S15, S16, S17, S18). These results highlighted that CREKA-GK8-QC exhibited better targeting and retention effects than CERAK-GK8-QC in subcutaneous breast tumors.

### Ex vivo* NIR fluorescence imaging of lung micro-metastasis through CREKA-GK8-QC*

**Fig. 5:**
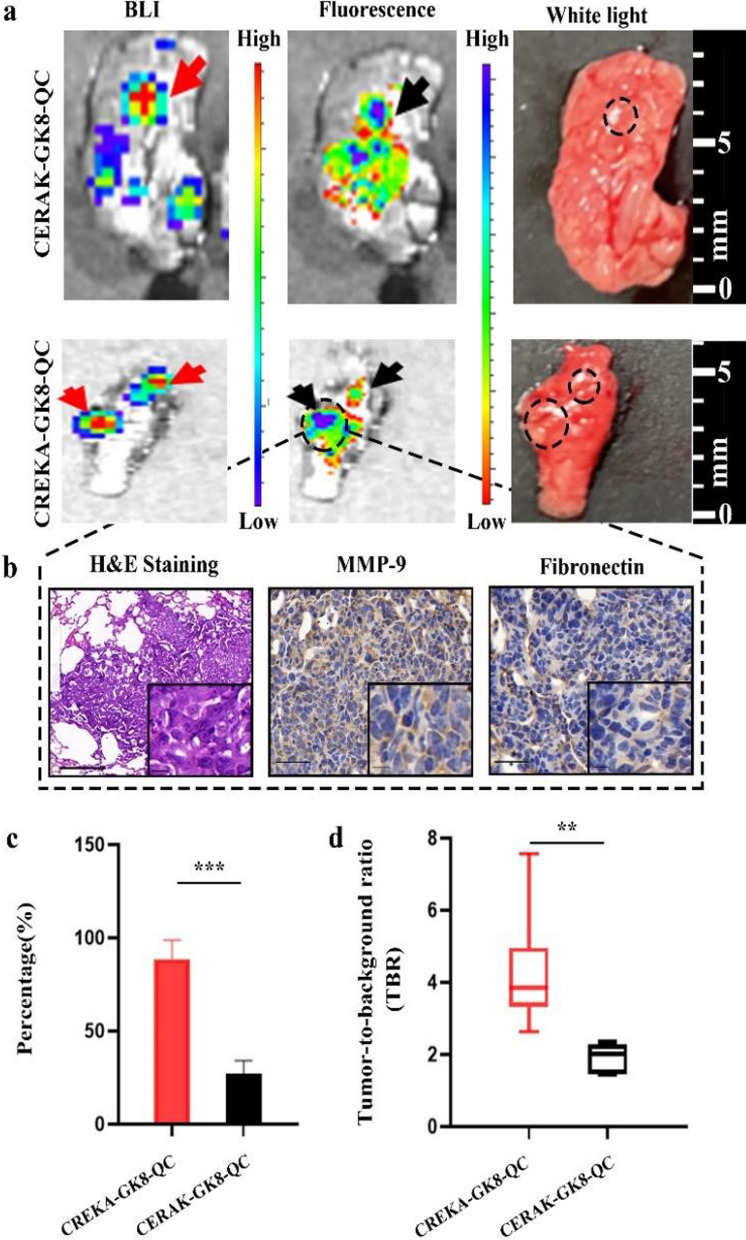
Ex vivo NIR fluorescence imaging of metastatic breast cancer lesions of lungs through CREKA-GK8-QC. **a**. Ex vivo BLI images, fluorescence images, and white light images of metastatic lungs at 24 h post-injection of CREKA-GK8-QC or CERAK-GK8-QC. The red and black arrows indicated the lightened-up metastatic lesions of lungs. **b**. H&E and immunohistochemical staining was utilized to confirm the true-positive metastatic lesion. Scale bar 50 µm. Inset: enlarged images of the metastasis lesions (scale bar 10 µm). **c**. Analysis of percentage of detection rate of metastatic lesions by CREKA-GK8-QC or CERAK-GKE-QC, respectively. Data represented as the mean ± SD, n = 3, ***p < 0.001. **d**. TBR of each lightened-up metastatic lesions by CREKA-GK8-QC or CERAK-GKE-QC. Data represented as the mean ± SD, n = 3, **p < 0.01

To further investigate the tumor-targeting effects of CREKA-GK8-QC on breast tumor metastatic lesions, NIR fluorescence imaging was applied to breast cancer lung metastasis mouse models. As shown in Additional file 1: Fig. S19, BLI detected the location of metastatic lesions in the lungs, indicating successful establishment of lung metastasis tumor model. NIR fluorescence imaging was performed at different time points after the injection of the imaging probe. For the in vivo observation, no obvious fluorescence signal of metastatic lesions between the surrounding tissue for mice treated with CREKA-GK8-QC was monitored, as well as with CERAK-GK8-QC (Additional file 1: Fig. S20). After in vivo observations, BLI and FMI of the ex vivo lungs with metastatic lesions and major organs were conducted (Additional file 1: Fig. S21). The fluorescence signals of the lungs with metastatic lesions were significantly enhanced in the CREKA-GK8-QC group compared with those in the control CERAK-GK8-QC group. Importantly, the colocalization of the BLI and FMI signals provided evidence of the high specificity and accuracy of the probe in detecting micro-metastasis of the lung (nearly 1–2 mm in diameter) (Fig. 5a).

Statistical analysis demonstrated that the average radiant efficiency of the lungs with metastatic lesions in the CREKA-GK8-QC group was almost 2 folds higher than that of the control CERAK-GK8-QC group (***p < 0.001, Additional file 1: Fig. S22). Furthermore, all metastatic lesions in the lungs detected by CREKA-GK8-QC were confirmed by BLI results, as well as ex vivo histological analysis (Fig. 5b). A total of 11 metastatic lesions, about 84.6% were successfully detected, which was nearly 3.4 times higher rate of metastatic lesions detection than CERAK-GK8-QC (25%, ***p < 0.001, Fig. 5c). By statistical calculation, the TBR in the CREKA-GK8-QC targeted group was 3.85 ± 1.45 at 24 h, which was 2 times higher than that in the CERAK-GK8-QC control group (1.92 ± 0.38, **p < 0.01, Fig. 5d). These results indicate that CREKA-GK8-QC possesses specific tumor-targeting abilities for ex vivo identification of cancer metastatic lesions, which could serve as a complementary tool for conventional intraoperative histological analysis.

For biosafety analysis, we performed H&E staining of the major organs, as well as liver and renal function assessment. No abnormalities were observed in the major organs, such as the heart, liver, spleen, lung, kidney, and intestines (Additional file 1: Fig. S23). In addition, alanine transaminase (ALT), aspartate transaminase (AST), and alkaline phosphatase (ALP) levels were assessed for liver function. To assess renal function, serum creatinine (Scr) and blood urea nitrogen (BUN) levels were measured. These data demonstrated that there were no differences between the two groups and the healthy normal mouse group, indicating that our probe had no obvious toxic effect on liver and kidney function (Additional file 1: Fig. S24).

### Comparison the therapeutic efficacy between CREKA-GK8-QC fluorescence image-guided surgery and conventional white light surgery

**Fig. 6:**
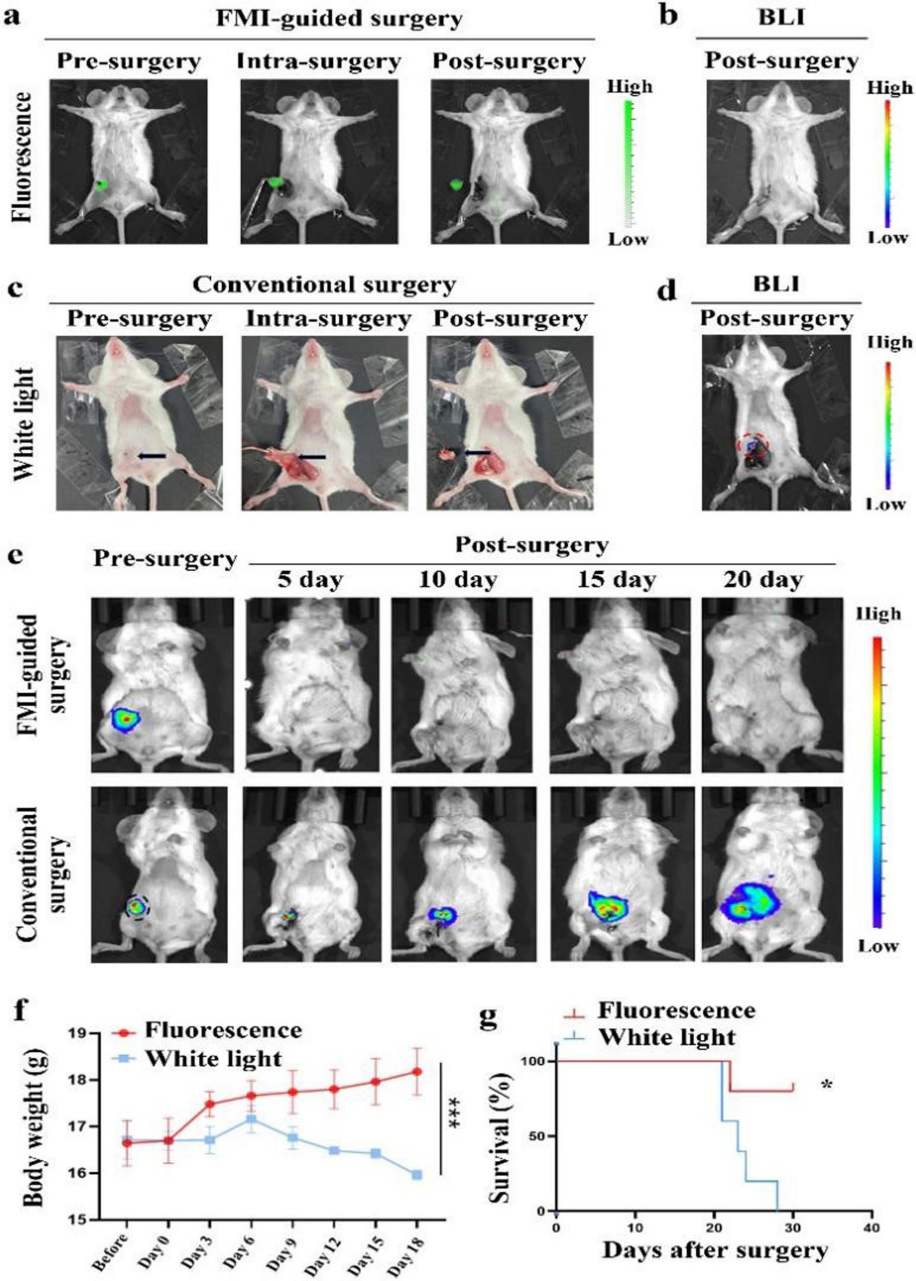
Fluorescence image-guided surgery with CREKA-GK8-QC and evaluation of tumor residuals (n = 5). **a**. Fluorescence images of orthotopic 4T1 breast tumor bearing mice treated with CREKA-GK8-QC-guided surgery at pre-, intra-, and post-surgery time. **b**. Tumor residuals were evaluated by BLI after fluorescence-guided surgery. **c**. White light images of orthotopic 4T1 breast tumor bearing mice treated with conventional surgery at pre-, intra-, and post-surgery time. **d**. Tumor residuals were further evaluated by BLI and the red blotted circle indicated the location of tumor residuals. **e.** BLI images of mice treated with fluorescence-guided surgery and conventional white light surgery at different time points before and of 20 days. **f.** Body weight examination after mice treated with fluorescence-guided surgery and conventional white light surgery from 0 to 18 days. Data represented as the mean ± SD, n = 5, ***p < 0.001. **g**. Comparison of survival rate with fluorescence image-guided surgery and conventional white light surgery. *p < 0.05

Owing to the sufficient TBR provided by the CREKA-GK8-QC, we further investigated its potential application for real-time intraoperative surgical guidance. CREKA-GK8-QC-guided resection of breast tumors was performed under a stereo fluorescence microscope 12 h after intravenous injection of CREKA-GK8-QC. NIRF imaging was performed pre-, intra-, and post-surgery to evaluate the tumor residuals (Fig. 6a). Conventional white light surgery was also performed, and white light images at pre-, intra-, and post-surgery times were recorded (Fig. 6c). Complete resection of tumor tissues was achieved in the CREKA-GK8-QC-guided surgery group, which was confirmed by BLI (Fig. 6b). However, small tumor residuals were detected after conventional white light surgery (Fig. 6d), which were validated by histological analysis (Additional file 1: Fig. S25). Moreover, in the fluorescence-guided surgery group, no obvious BLI signals or significant body weight loss appeared within 20 days after the operation. In the conventional white light surgery group, the residual lesions were detectable through BLI signals gradually increased from days 5 to 20 (Fig. 6e), and obvious body weight loss was observed at day 6 (Fig. 6f). Moreover, Kaplan–Meier analysis demonstrated that compared with the conventional white light surgery, the survival rate increased from 0 to 80% within 30 days after CREKA-GK8-QC-guided fluorescence surgery (*p < 0.05) (Fig. 6g). The above results were attributed to the high recurrence rate (100%) the in the conventional white light surgery group, which was much higher than that (20%) in CREKA-GK8-QC-guided surgery group (Additional file 1: Table S1). These results highlighted the important role for CREKA-GK8-QC in intraoperative tumor detection, which significantly decreased tumor recurrence and improved the survival rate compared with conventional white light surgery.

## Discussion

In our study, a novel fibronectin-targeted and MMP-9-activatable imaging probe, CREKA-GK8-QC, was synthesized to detect orthotopic and metastatic breast cancer, as well as fluorescence image-guided surgical resection. Supported by in vivo preclinical 4T1 orthotopic and metastatic tumor mouse models and ex vivo image analysis, CREKA-GK8-QC possessed great potential for intraoperative image-guided surgery for breast cancer. To our knowledge, our developed dual-targeted fluorescence imaging probe can specifically target MMP-9 and fibronectin and enable the intraoperative detection and surgery of breast cancer with high specificity.

In various preclinical studies, MMP-9 and fibronectin have been identified as specific imaging biomarkers for breast cancer [23, 27]. MMP-9 affects remodeling of the extracellular matrix and cancer invasiveness [28]. As an essential product of the tumor interstitial inflammatory reaction, fibronectin is significantly overexpressed and correlates well with tumor fibrosis and infiltration [29]. MMP-9-activatable imaging probes have been successfully applied for specific imaging of cancer [30] and targeted anticancer drug delivery systems [25]. Fibronectin-targeted CREKA-based imaging probes have been widely applied in cancer diagnosis and treatment [31, 32]. However, a dual-targeting fluorescence imaging probe for MMP-9 and fibronectin has not yet been reported. Multi-targets targeting imaging probes can enhance the specificity for cancer lesion detection, yielding a stronger selective efficiency, and lower background signal for cancer [33]. This advantage could support surgical decision-making in the clinic, including facilitating the precise cancer diagnosis, complete resection and reducing iatrogenic injury intraoperatively. Therefore, CREKA-GK8-QC may represent an interesting dual-targeting strategy that allows for the accurate assessment of both primary and metastatic breast cancers, which promotes the development of clinical-used multifunctional fluorescence probes with improved tumor-targeting efficacy [34, 35].

To reduce the risk of breast cancer recurrence, there is an urgent need to develop a new contrast agent for intraoperative tumor delineation. Optical imaging, such as FMI, is a promising approach for identifying residual tumors with millimeters of penetration at cellular resolution [36, 37]. Currently, most studies only focus on the roles of MMP-9-activable imaging probes for cancer imaging, and seldom utilized them for intraoperative surgical guidance. We first assessed the important role of CREKA-GK8-QC in fluorescence-guided surgery for breast cancer. This study supports the use of a dual-targeted imaging probe to delineate the tumor boundary from the surrounding normal tissues during tumor resection. CREKA-GK8-QC exhibited more specific targeting and longer retention of fluorescence signals at the tumor sites than CERAK-GK8-QC. The BLI imaging of 4T1 tumors revealed that FMI-intraoperative guidance with CREKA-GK8-QC enabled a more precise resection of tumors with no detectable tumor residuals and lower recurrence rate (20%). No adverse events were observed postoperatively, and the fluorescence image-guided surgery group achieved longer survival than the conventional surgery group. For years, complete tumor resection has stood for the primary goal of breast cancer surgery. The above results confirmed the significant roles of FMI for guiding surgeons removing otherwise undetectable cancer lesions to achieve precision surgery. Thus, this type of dual-targeted imaging probe holds a promising application for further clinical surgical guidance and improving prognosis [38].

Lung metastasis is an essential factor for advanced-stage breast cancer as it directly determines the survival rate [39]. Current conventional imaging techniques have several limitations for intraoperative real-time detection, including low detection rate, high cost, and inconvenience. Therefore, finding sensitive and safe diagnostic tools for intraoperative metastasis detection are urgently needed. Our CREKA-GK8-QC probe identified most metastatic tumors (80%), as corroborated by histopathology. More importantly, CREKA-GK8-QC offered a useful platform for the early diagnosis of micro-metastasis (nearly 1 mm in diameter) with high imaging contrast, which may improve the detection rate compared with traditional white light. These results demonstrate the great potential of CREKA-GK8-QC in the further sensitive intraoperative detection of micro-metastasis and assisting pathological analysis.

There are some limitations to our study. The fluorescent dye Cy5.5 lacked significant penetration depth to ensure sufficient fluorescence signals passing through the orthotopic deep tumors. Hence, utilizing the fluorescence in the NIR-II region may improve the image depth and facilitate more disease foci detection [40, 41]. Simultaneously, through utilizing multimodality imaging as combined PET or emerging magnetic particle imaging may facilitate the longitudinal assessment on in vivo targeting properties and pharmaceutics of imaging probes [40]. Additionally, designing advanced imaging probes (e.g., higher tumor-to-background ration, quantitative properties) and technologies (e.g., multi-channel imaging, cutting-edge imaging system) may also improve the diagnosis rate of breast cancer and long-term survival benefits [38].

## Conclusions

In this study, we report a fibronectin-targeting and MMP-9-activatable imaging probe (CREKA-GK8-QC) used for specific fluorescence imaging of breast tumors (including the primary malignancy and lung metastatic foci) and image-guided complete resection of breast tumors. MMP-9 and fibronectin are both highly expressed in the breast cancer TME; Therefore, our imaging and therapeutic strategy can enable real-time detection of 4T1 breast primary and micro-metastatic lesions and image-guided cancer surgery. CREKA-GK8-QC exhibited more specific and longer retention of fluorescence signals than the control CERAK-GK8-QC imaging probe, and facilitated the complete resection of breast tumors and better prognosis. This study provides a foundation for future translational clinical trials. In future studies, we will develop fluorescence dye-labeled imaging probes with better penetration depth or combined with other imaging modalities to achieve more specific and sensitive in vivo tumor lesion identification, which may pave the way for developing turn-on imaging probes for clinical cancer imaging and fluorescence image-guided surgery of tumor lesions.

## Supplementary Information

**Additional file 1: Fig. S1** IHC staining of MMP-9 in orthotopic breast cancer and normal breast tissues. High levels of MMP-9 expression were detected by IHC staining in orthotopic breast lesions from 4T1 tumor-bearing mice (scale bar: 50 μm). Relative lower expression levels of MMP-9 were detected in normal breast tissues (scale bar: 50 μm). **Fig. S2** IHC staining of fibronectin in orthotopic breast cancer and normal breast tissues. High levels of fibronectin expression were detected by IHC staining in orthotopic lesions from 4T1 tumor-bearing mice (scale bar: 50 μm). Relative lower expression levels of fibronectin were detected in normal breast tissues (scale bar: 50 μm). **Fig. S3** IHC staining of MMP-9 in lung metastasis and normal lung tissues. High levels of MMP-9 expression were detected by IHC staining in metastatic lung lesions from 4T1 tumor-bearing mice (scale bar: 50 μm). Relative lower expression levels of MMP-9 were detected in normal lung tissues (scale bar: 50 μm). **Fig. S4** IHC staining of fibronectin in lung metastasis and normal lung tissues. High levels of fibronectin expression were detected by IHC staining in metastatic lung lesions from 4T1 tumor-bearing mice (scale bar: 50 μm). Relative lower expression levels of fibronectin were detected in normal lung tissues (scale bar: 50 μm). **Fig. S5** Examination of MMP-9 expression on murine 4T1 breast cancer. The expression level of MMP-9 in 4T1 breast tumor and normal breast tissues examined by Western Blot analysis. Densitometric analysis of expression level of MMP-9 normalized to that of GAPDH. Data represented as the mean ± SD, n=3, **p<0.01. **Fig. S6** Examination of fibronectin expression on murine 4T1 breast cancer. The expression level of fibronectin in 4T1 breast tumor and normal murine breast tissues examined by Western Blot analysis. Densitometric analysis of expression level fibronectin normalized to that of GAPDH. Data represented as the mean ± SD, n=3, **p<0.01. **Fig. S7** HR-MS spectra of Sulfo Cy5.5-GK8-QSY21. **Fig. S8** HR-MS spectra of CREKA-GK8-QC. **Fig. S9** HR-MS spectra of CERAK-GK8-QC. **Fig. S10** IF staining of subcutaneous breast tumors. Analysis of MMP-9 or fibronectin expression and distribution of targeted CREKA-GK8-Cy5.5 and control CERAK-GK8-QC in 4T1 subcutaneous breast tumor sections, respectively. DAPI was used for staining nuclei. Pseudo-colors in the confocal images are assigned as follows: red, Cy5.5; green, MMP-9 or fibronectin; and blue, nucleus. Scale bar 50 µm. **Fig. S11** IF staining of lung metastasis. Analysis of MMP-9 or fibronectin expression and distribution of targeted CREKA-GK8-Cy5.5 and control CERAK-GK8-QC in metastatic lung sections, respectively. DAPI was used for staining nuclei. Pseudo-colors in the confocal images are assigned as follows: red, Cy5.5; green, MMP-9 or fibronectin; and blue, nucleus. Scale bar 50 µm. **Fig. S12** The cytotoxicity assays of different concentrations of fluorescence imaging probe CREKA-GK8-QC (0, 20, 40, 80, 100, and 200 μg/ml) were measured using CCK-8 with murine 4T1 cells. **Fig. S13** Ex vivo fluorescence intensities of tumors and major organs at 48 hours post-injection of CREKA-GK8-QC or CERAK-GK8-QC. Data represented as the mean ± SD, n=3, *p<0.05. **Fig. S14** BLI imaging of subcutaneous 4T1 breast tumors. BLI of subcutaneous 4T1 tumor bearing mice which was applied to detect the location of tumors. **Fig. S15** In vivo fluorescence imaging of subcutaneous 4T1 breast tumors. In vivo fluorescence images of subcutaneous breast cancer bearing mice injected with CREKA-GK8-QC or CERAK-GK8-QC (n=3) at different time points. The dotted black circles indicated the locations of orthotopic breast tumors. **Fig. S16** In vivo TBR at different time points. Quantification of in vivo fluorescence imaging TBR. Data represented as the mean ± SD, n=3, **p<0.01. **Fig. S17** Ex vivo fluorescence imaging of subcutaneous 4T1 breast tumors and major organs. Ex vivo fluorescence imaging of tumors and major organs, including liver, kidney, heart, spleen, lung, and intestine, at 48 hours post-injection of CREKA-GK8-QC or CERAK-GK8-QC. **Fig. S18** Fluorescence intensities of subcutaneous 4T1 breast tumors and major organs. Ex vivo fluorescence intensities of tumors and major organs at 48 hours post-injection of CREKA-GK8-QC or CERAK-GK8-QC. Data represented as the mean ± SD, n=3, *p<0.05. **Fig. S19** BLI imaging of metastatic lung lesions. BLI of lung metastasis in 4T1 tumor bearing mice which was applied to detect the location of metastatic lesions. **Fig. S20** In vivo fluorescence imaging of metastatic lung lesions. In vivo fluorescence images of metastatic breast cancer bearing mice injected with CREKA-GK8-QC or CERAK-GK8-QC (n=3) at different time points. The dotted black circles indicated the locations of metastatic lesions. **Fig. S21** Ex vivo BLI and fluorescence images of metastatic lung lesions 4T1 tumor bearing mice at 24 h post injection of CREKA-GK8-QC or CERAK-GK8-QC. **Fig. S22** Ex vivo fluorescence intensities of metastatic lungs and major organs at 24 hours post-injection of CREKA-GK8-QC or CERAK-GK8-QC. Data represented as the mean ± SD, n=3, ***p<0.001. **Fig. S23** Histologic analysis of major organs. HE staining images of major organs (heart, liver, spleen, lung, kidney, and spleen) from different groups. Scale bars: 50 μm. **Fig. S24** Liver and kidney function analysis. Liver function, including a. ALT, b. AST, and c. ALP and kidney function, including d. Scr and e. BUN, were all analyzed from different groups. **Fig. S25** H&E staining was utilized to confirm the tumor residuals. Scale bar 50 µm. Inset: enlarged images of the metastasis sections (scale bar 10 µm). **Table. S1** Local tumor recurrence after surgery.

## Data Availability

The data used and/or analyzed during this study are available from the corresponding author upon reasonable request.

## Abbreviations

BCS: Breast-conserving surgery
MI: Molecular imaging
FMI: Fluorescence molecular imaging
MMP: Matrix metalloproteinase
TNBC: Triple-negative breast cancer
TME: Tumor microenvironment
NIRF: Near infrared fluorescence
