# Supplementary material for: Fibronectin-targeting and metalloproteinase-activatable smart imaging probe for fluorescence imaging and image-guided surgery of breast cancer
Source: J Nanobiotechnology. 2023 Mar 28;21:112. doi: 10.1186/s12951-023-01868-5 (PMC10053476; doi:10.1186/s12951-023-01868-5)
Supplement: Supplementary file 1 — Additional file 1: Fig. S1 IHC staining of MMP-9 in orthotopic breast cancer and normal breast tissues. High levels of MMP-9 expression were detected by IHC staining in orthotopic breast lesions from 4T1 tumor-bearing mice (scale bar: 50 μm). Relative lower expression levels of MMP-9 were detected in normal breast tissues (scale bar: 50 μm). Fig. S2 IHC staining of fibronectin in orthotopic breast cancer and normal breast tissues. High levels of fibronectin expression were detected by IHC staining in orthotopic lesions from 4T1 tumor-bearing mice (scale bar: 50 μm). Relative lower expression levels of fibronectin were detected in normal breast tissues (scale bar: 50 μm). Fig. S3 IHC staining of MMP-9 in lung metastasis and normal lung tissues. High levels of MMP-9 expression were detected by IHC staining in metastatic lung lesions from 4T1 tumor-bearing mice (scale bar: 50 μm). Relative lower expression levels of MMP-9 were detected in normal lung tissues (scale bar: 50 μm). Fig. S4 IHC staining of fibronectin in lung metastasis and normal lung tissues. High levels of fibronectin expression were detected by IHC staining in metastatic lung lesions from 4T1 tumor-bearing mice (scale bar: 50 μm). Relative lower expression levels of fibronectin were detected in normal lung tissues (scale bar: 50 μm). Fig. S5 Examination of MMP-9 expression on murine 4T1 breast cancer. The expression level of MMP-9 in 4T1 breast tumor and normal breast tissues examined by Western Blot analysis. Densitometric analysis of expression level of MMP-9 normalized to that of GAPDH. Data represented as the mean ± SD, n=3, **p<0.01. Fig. S6 Examination of fibronectin expression on murine 4T1 breast cancer. The expression level of fibronectin in 4T1 breast tumor and normal murine breast tissues examined by Western Blot analysis. Densitometric analysis of expression level fibronectin normalized to that of GAPDH. Data represented as the mean ± SD, n=3, **p<0.01. Fig. S7 HR-MS spectra of Sulfo Cy5.5-G [file 12951_2023_1868_MOESM1_ESM.docx]

**Fibronectin-targeting and metalloproteinase-activatable smart imaging probe for fluorescence imaging and image-guided surgery of breast cancer**

Zhongquan Cheng^1, 2, #^, Yushen Jin^3, #^, Jiaqian Li^4^, Guangyuan Shi^5^, Leyi Yu^6^, Bing Shao^3,*^, Jie Tian^2, 8, *^, Yang Du^2, 7, *^, Zhu Yuan^1, *^

^1^Department of General Surgery, Capital Medical University, Beijing Friendship Hospital, Beijing, 100050, China

^2^CAS Key Laboratory of Molecular Imaging, Beijing Key Laboratory of Molecular Imaging, Institute of Automation, Chinese Academy of Sciences, Beijing, 100190, China

^3^Beijing Key Laboratory of Diagnostic and Traceability Technologies for Food Poisoning, Beijing Center for Disease Prevention and Control, Beijing, 100013, China

^4^School of Biological Science and Medical Engineering, Beihang University, Beijing, 100083, China

^5^University of Science and Technology of China, Anhui, 230026, China

^6^Haidian Section of Peking University Third Hospital, Beijing, 100080, China

^7^University of Chinese Academy of Sciences, Beijing, 100080, China

^8^Beijing Advanced Innovation Center for Big Data-Based Precision Medicine, School of Medicine Science and Engineering, Beihang University, Beijing, 100191, China

^#^These authors contributed equally to this work

*Corresponding authors: Jie Tian, email: tian@ieee.org; Tel: +86-10-82628760; Fax: +86-10-62527995, Beijing Advanced Innovation Center for Big Data-Based Precision Medicine, School of Medicine Science and Engineering, Beihang University, Beijing, 100191, China; Yang Du, email: yang.du@ia.ac.cn, CAS Key Laboratory of Molecular Imaging, Institute of Automation, Beijing, Chinese Academy of Sciences, 100190, China; Bing Shao, email: shaobingch@sina.com, Beijing Key Laboratory of Diagnostic and Traceability Technologies for Food Poisoning, Beijing Center for Disease Prevention and Control, Beijing, 100013, China; Zhu Yuan, email: BJFH_yz_paper@163.com, Capital Medical University, Beijing Friendship Hospital, Beijing, 100050, China.

**Experimental materials and methods**

***In vivo* biosafety assay**

Six-week-old female Balb/c mice (Vital River Laboratories, Beijing, China) were intravenously injected with 100 μL CREKA-GK8-QC or CERAK-GK8-QC. In addition, another group of mice was treated with the same volume of PBS as the control group (n = 3). After 48 h, all mice were euthanized, and their major organs (liver, lung, spleen, heart, kidney, and intestine) and serum were collected for biosafety evaluation. Liver function indicators (alanine aminotransferase, ALT; aspartate aminotransferase, AST; ALP: alkaline phosphatase), and biochemical indicators of kidney function (creatinine, CREA; blood urea nitrogen, BUN) were examined using a blood biochemistry autoanalyzer (HITACHI 7600, Japan). The major organs were wax-embedded and sliced into 4-μm sections, followed by staining with hematoxylin and eosin (H&E). Images were captured using a PANNORAMIC MIDI II scanner (3DHISTECH, Hungary).


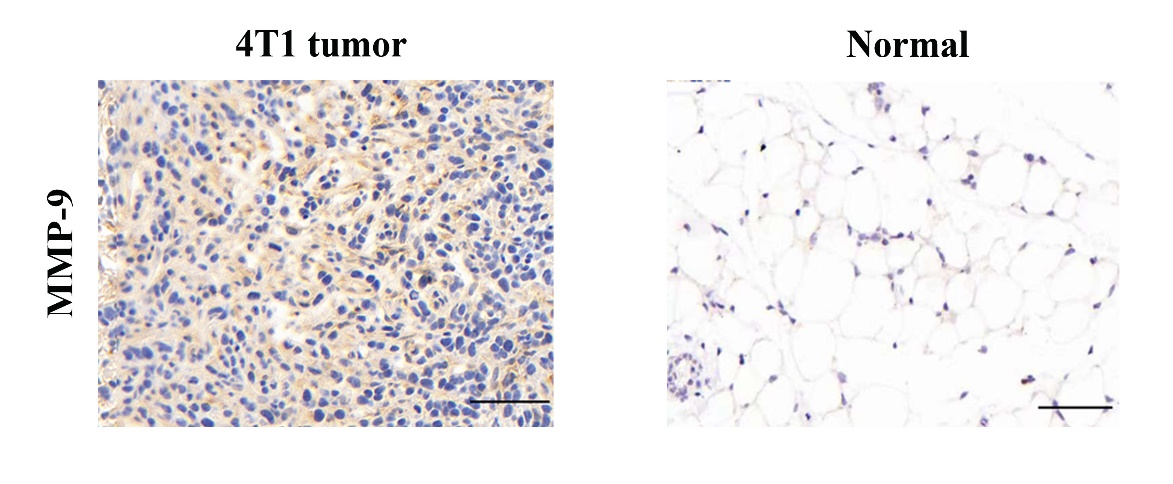


**Fig. S1** IHC staining of MMP-9 in orthotopic breast cancer and normal breast tissues. High levels of MMP-9 expression were detected by IHC staining in orthotopic breast lesions from 4T1 tumor-bearing mice (scale bar: 50 μm). Relative lower expression levels of MMP-9 were detected in normal breast tissues (scale bar: 50 μm).


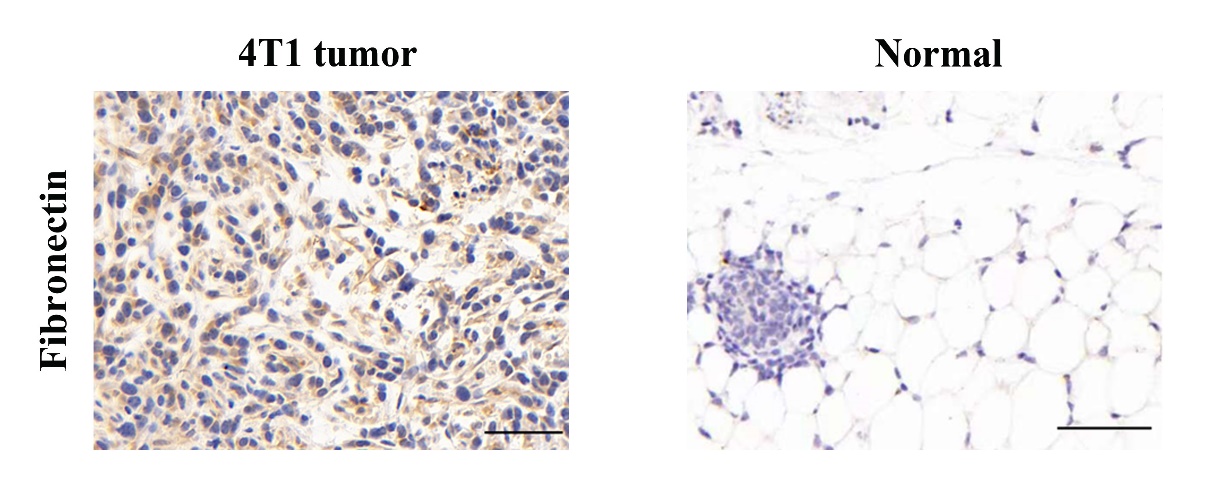


**Fig. S2** IHC staining of fibronectin in orthotopic breast cancer and normal breast tissues. High levels of fibronectin expression were detected by IHC staining in orthotopic lesions from 4T1 tumor-bearing mice (scale bar: 50 μm). Relative lower expression levels of fibronectin were detected in normal breast tissues (scale bar: 50 μm).


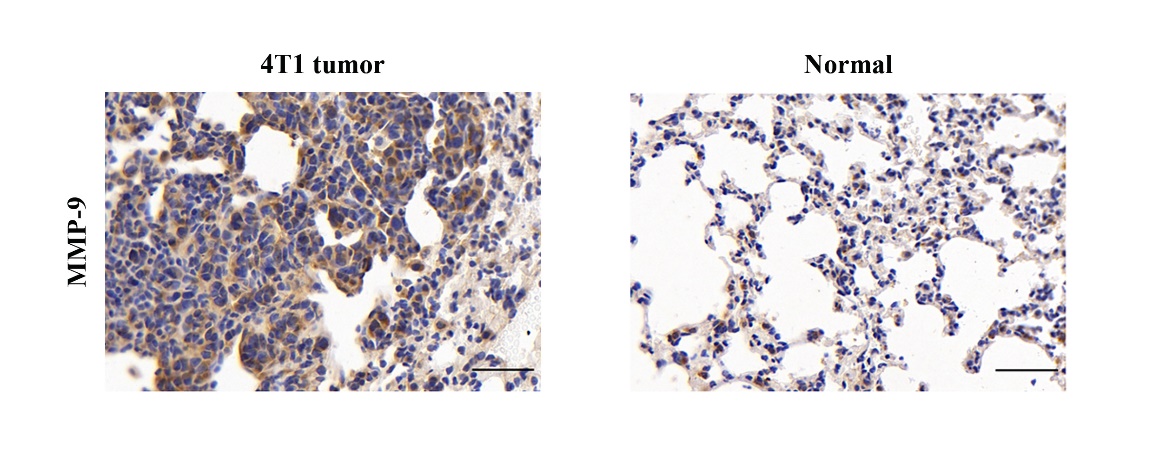


**Fig. S3** IHC staining of MMP-9 in lung metastasis and normal lung tissues. High levels of MMP-9 expression were detected by IHC staining in metastatic lung lesions from 4T1 tumor-bearing mice (scale bar: 50 μm). Relative lower expression levels of MMP-9 were detected in normal lung tissues (scale bar: 50 μm).


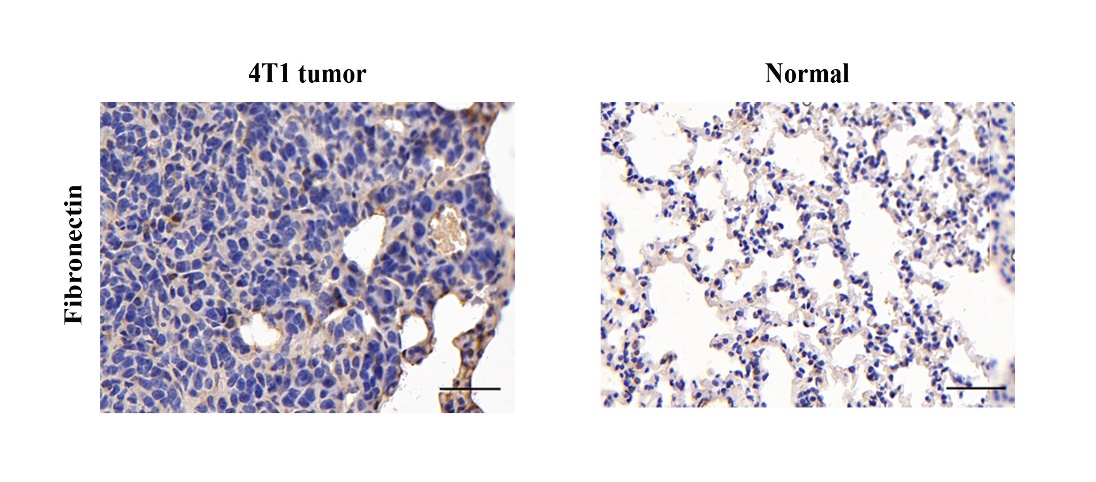


**Fig. S4** IHC staining of fibronectin in lung metastasis and normal lung tissues. High levels of fibronectin expression were detected by IHC staining in metastatic lung lesions from 4T1 tumor-bearing mice (scale bar: 50 μm). Relative lower expression levels of fibronectin were detected in normal lung tissues (scale bar: 50 μm).


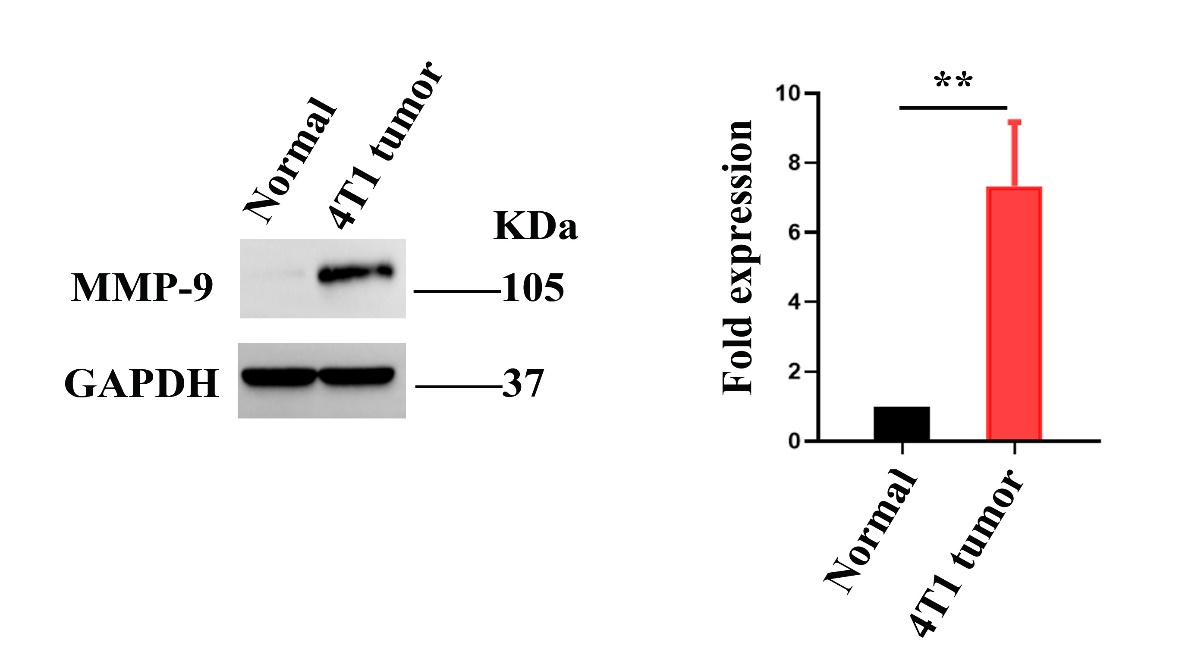


**Fig. S5** Examination of MMP-9 expression on murine 4T1 breast cancer. The expression level of MMP-9 in 4T1 breast tumor and normal breast tissues examined by Western Blot analysis. Densitometric analysis of expression level of MMP-9 normalized to that of GAPDH. Data represented as the mean ± SD, n=3, **p<0.01.


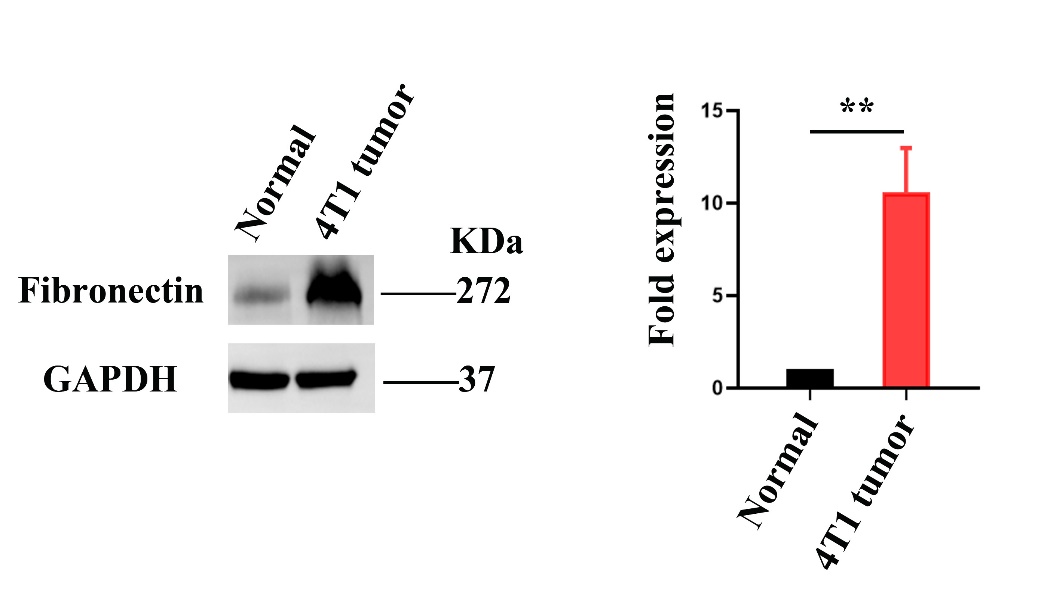


**Fig. S6** Examination of fibronectin expression on murine 4T1 breast cancer. The expression level of fibronectin in 4T1 breast tumor and normal murine breast tissues examined by Western Blot analysis. Densitometric analysis of expression level fibronectin normalized to that of GAPDH. Data represented as the mean ± SD, n=3, **p<0.01.


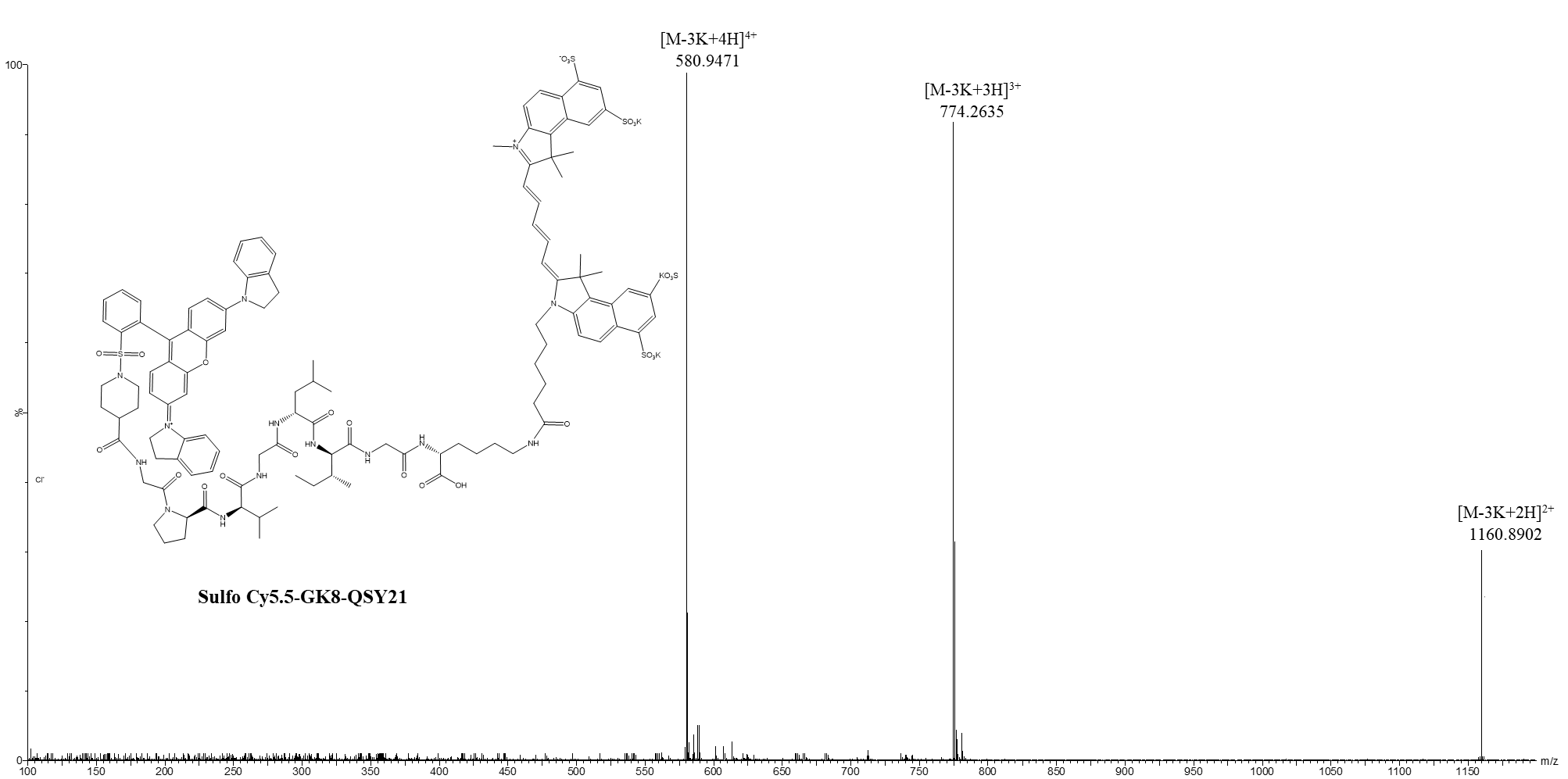


**Fig. S7** HR-MS spectra of Sulfo Cy5.5-GK8-QSY21.


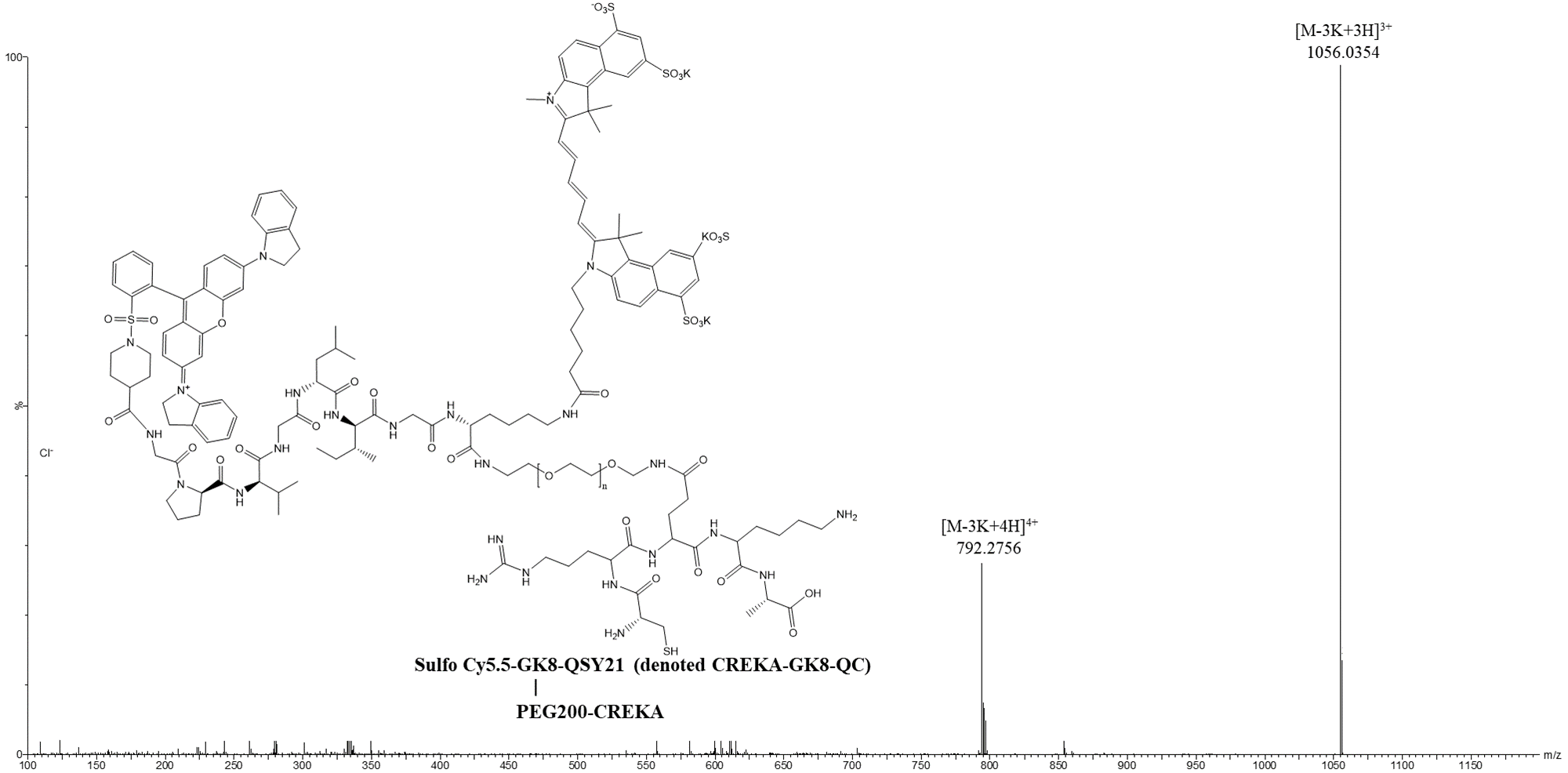


**Fig. S8** HR-MS spectra of CREKA-GK8-QC.


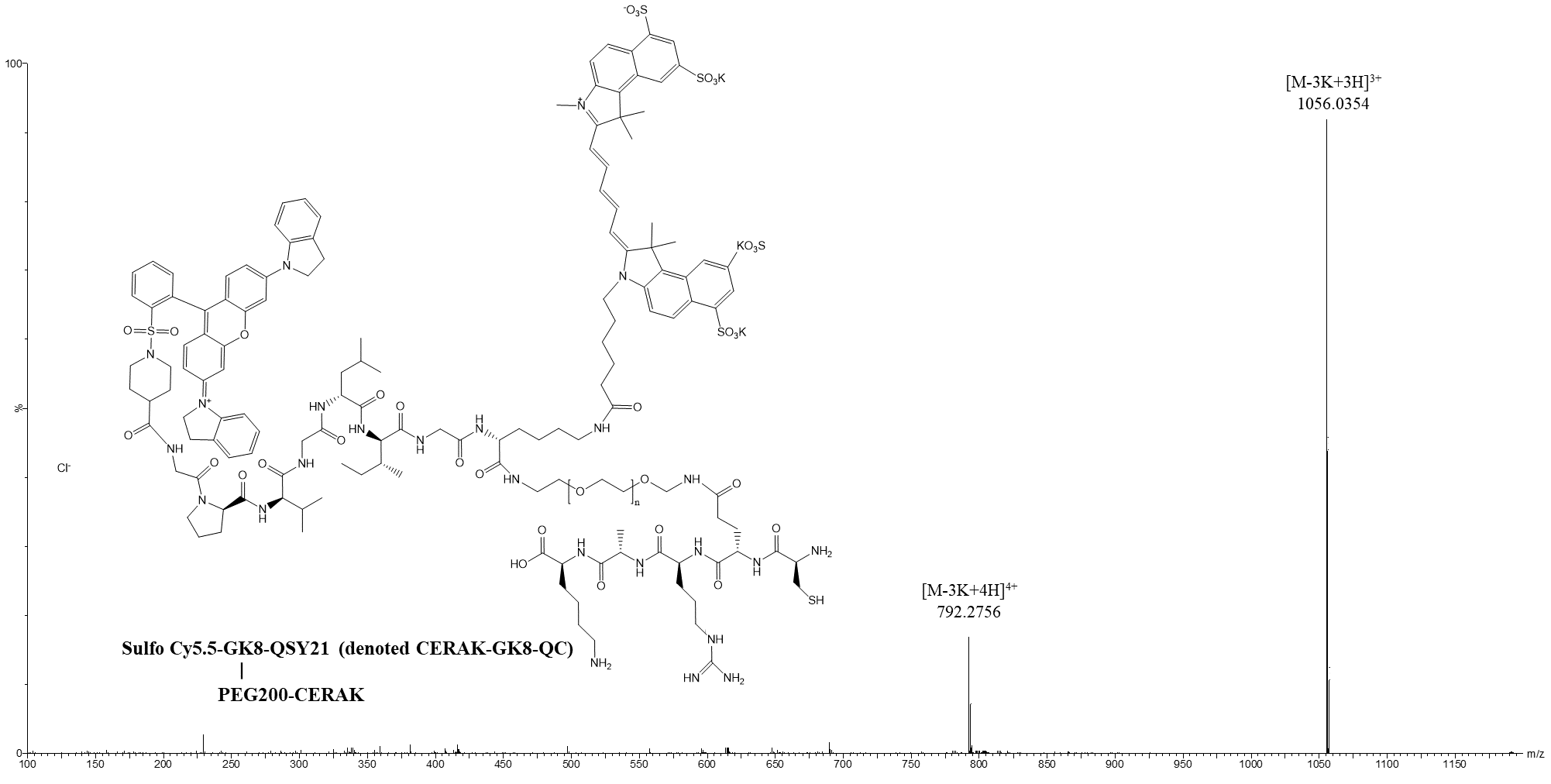


**Fig. S9** HR-MS spectra of CERAK-GK8-QC.


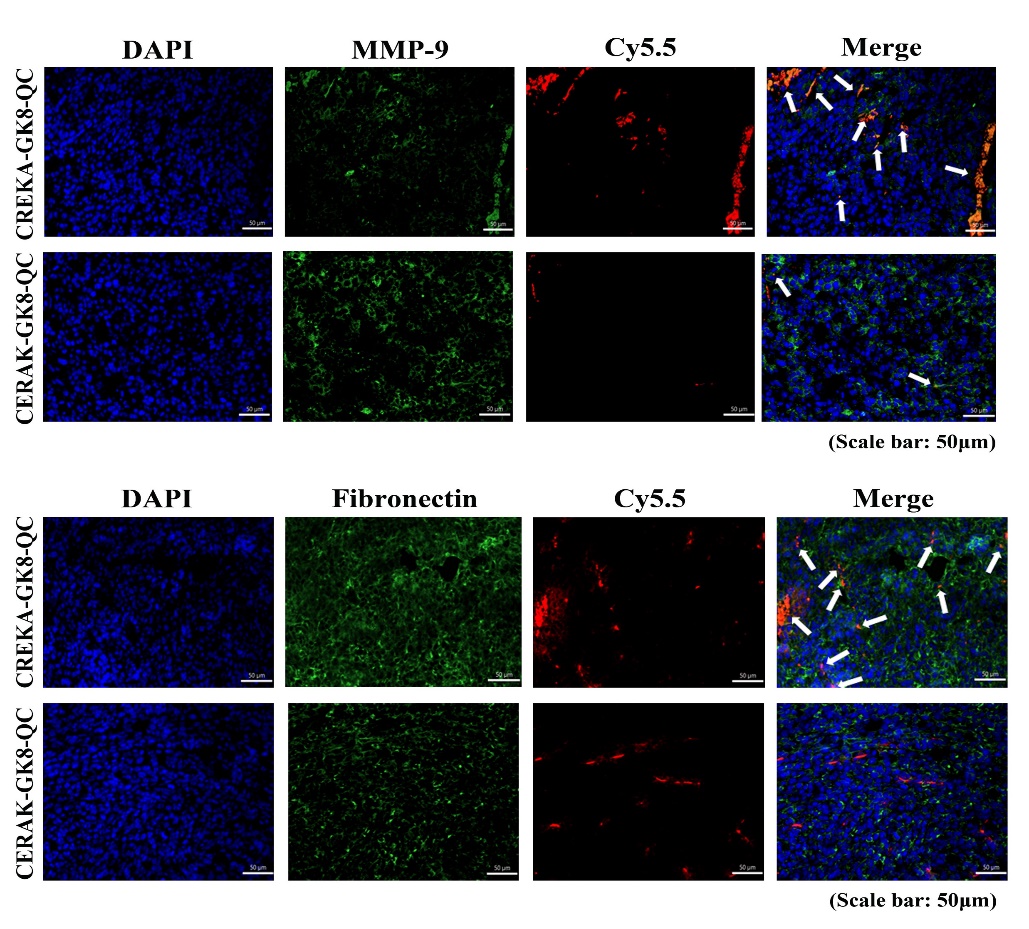


**Fig. S10** IF staining of subcutaneous breast tumors. Analysis of MMP-9 or fibronectin expression and distribution of targeted CREKA-GK8-Cy5.5 and control CERAK-GK8-QC in 4T1 subcutaneous breast tumor sections, respectively. DAPI was used for staining nuclei. Pseudo-colors in the confocal images are assigned as follows: red, Cy5.5; green, MMP-9 or fibronectin; and blue, nucleus. Scale bar 50 µm**.**


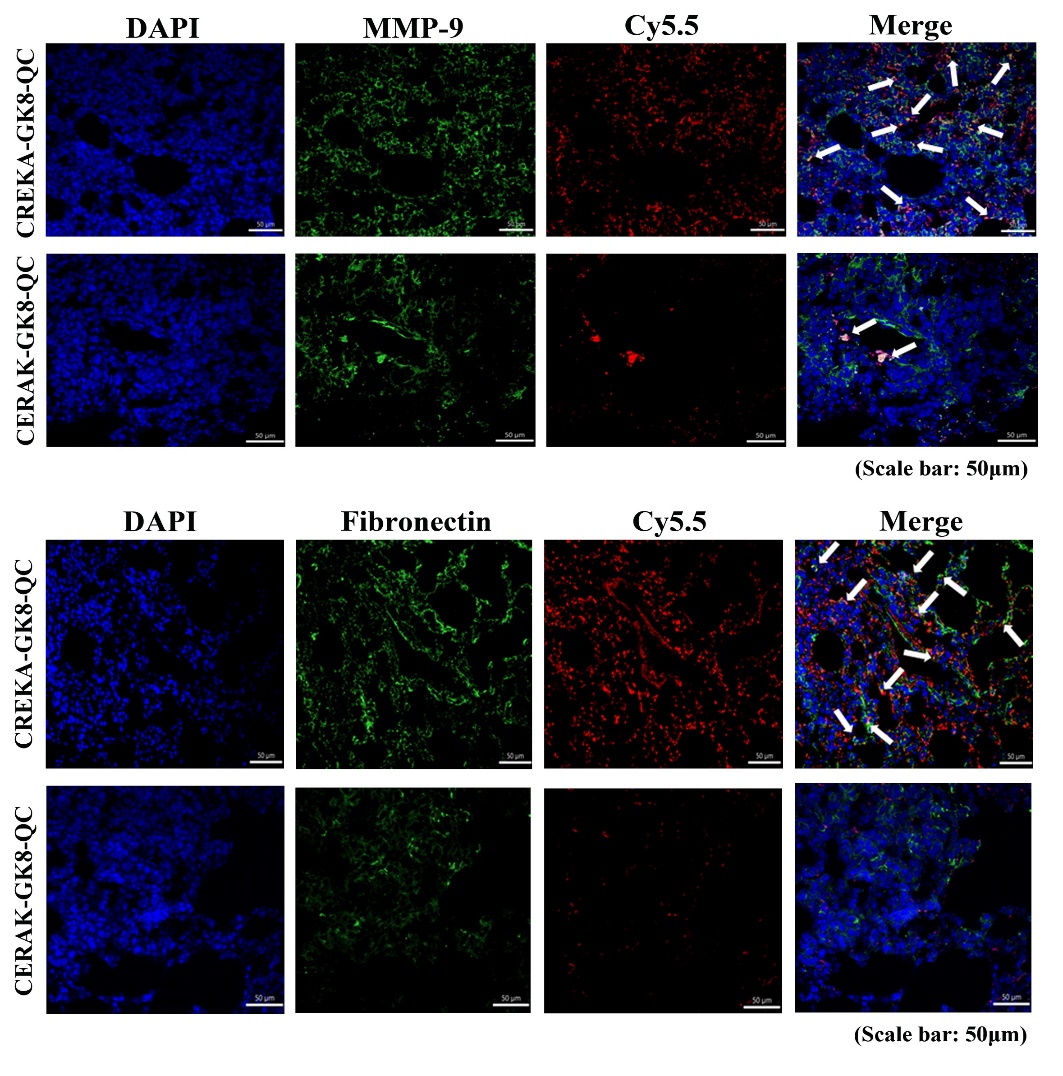


**Fig. S11** IF staining of lung metastasis. Analysis of MMP-9 or fibronectin expression and distribution of targeted CREKA-GK8-Cy5.5 and control CERAK-GK8-QC in metastatic lung sections, respectively. DAPI was used for staining nuclei. Pseudo-colors in the confocal images are assigned as follows: red, Cy5.5; green, MMP-9 or fibronectin; and blue, nucleus. Scale bar 50 µm**.**


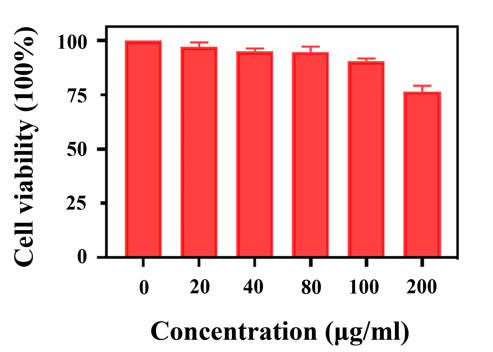


**Fig. S12** The cytotoxicity assays of different concentrations of fluorescence imaging probe CREKA-GK8-QC (0, 20, 40, 80, 100, and 200 μg/ml) were measured using CCK-8 with murine 4T1 cells.


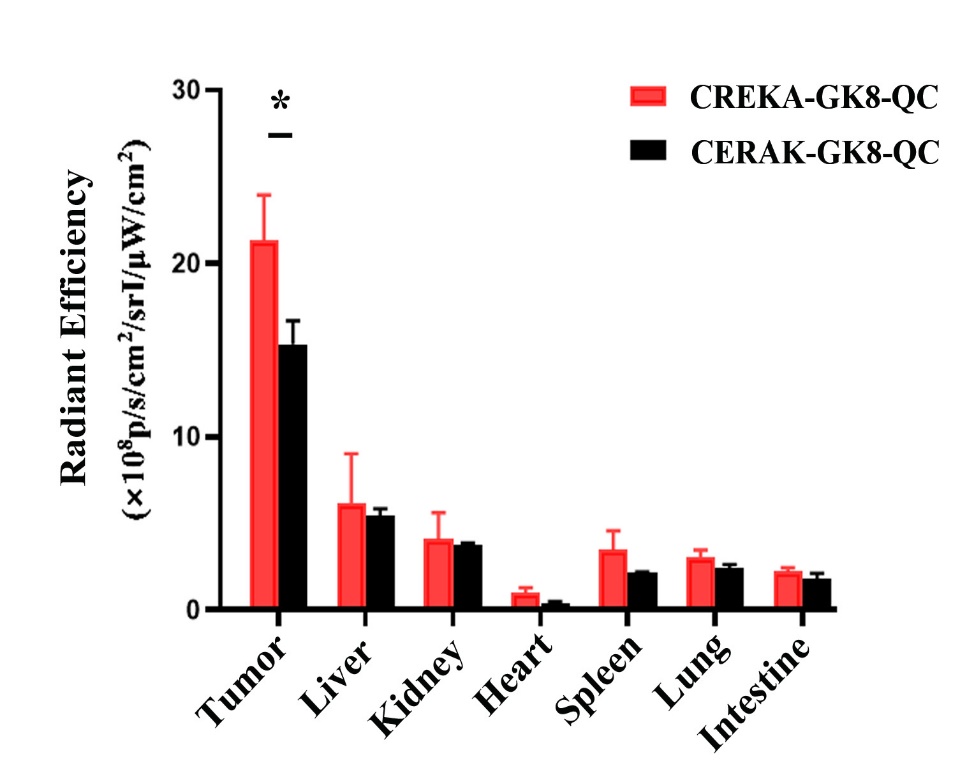


**Fig. S13** *Ex vivo* fluorescence intensities of tumors and major organs at 48 hours post-injection of CREKA-GK8-QC or CERAK-GK8-QC. Data represented as the mean ± SD, n=3, *p<0.05.


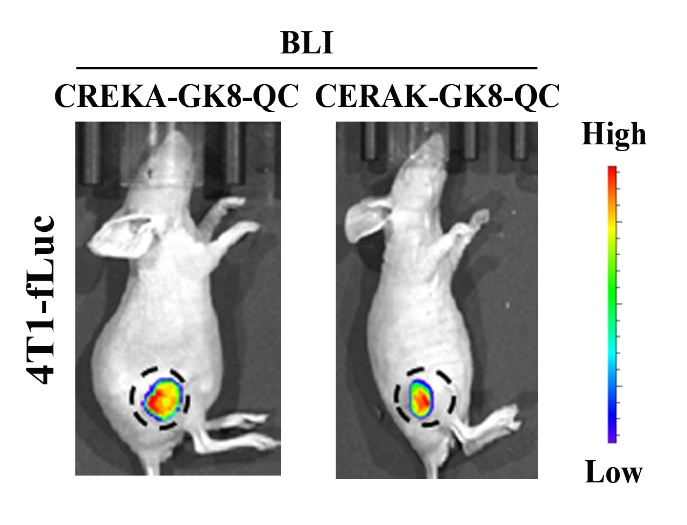


**Fig. S14** BLI imaging of subcutaneous 4T1 breast tumors. BLI of subcutaneous 4T1 tumor bearing mice which was applied to detect the location of tumors.


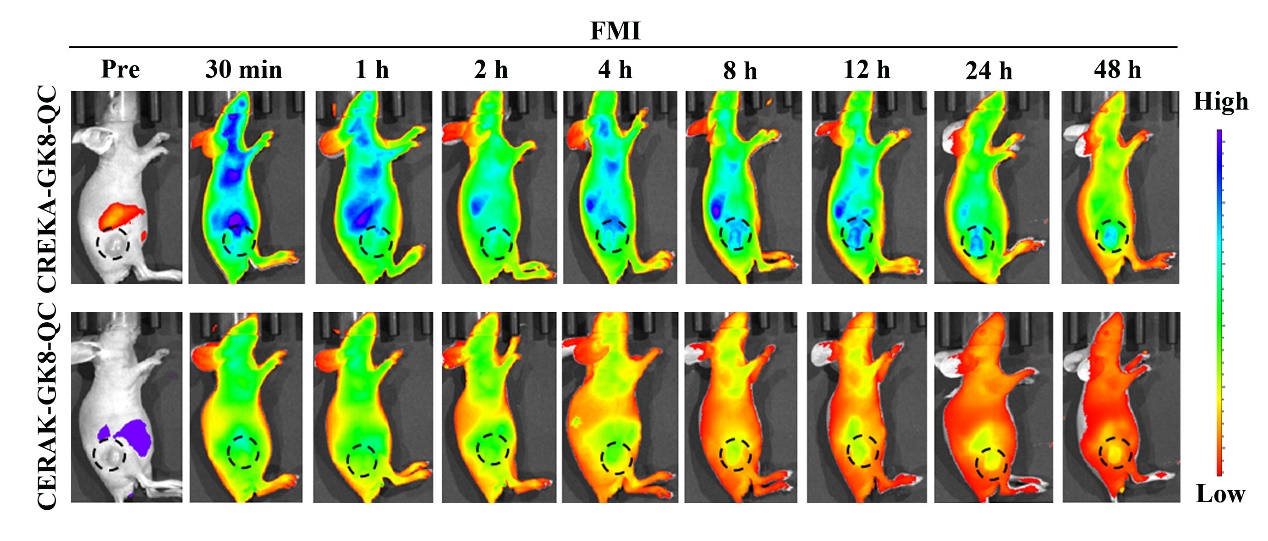


**Fig. S15** *In vivo* fluorescence imaging of subcutaneous 4T1 breast tumors. *In vivo* fluorescence images of subcutaneous breast cancer bearing mice injected with CREKA-GK8-QC or CERAK-GK8-QC (n=3) at different time points. The dotted black circles indicated the locations of orthotopic breast tumors.


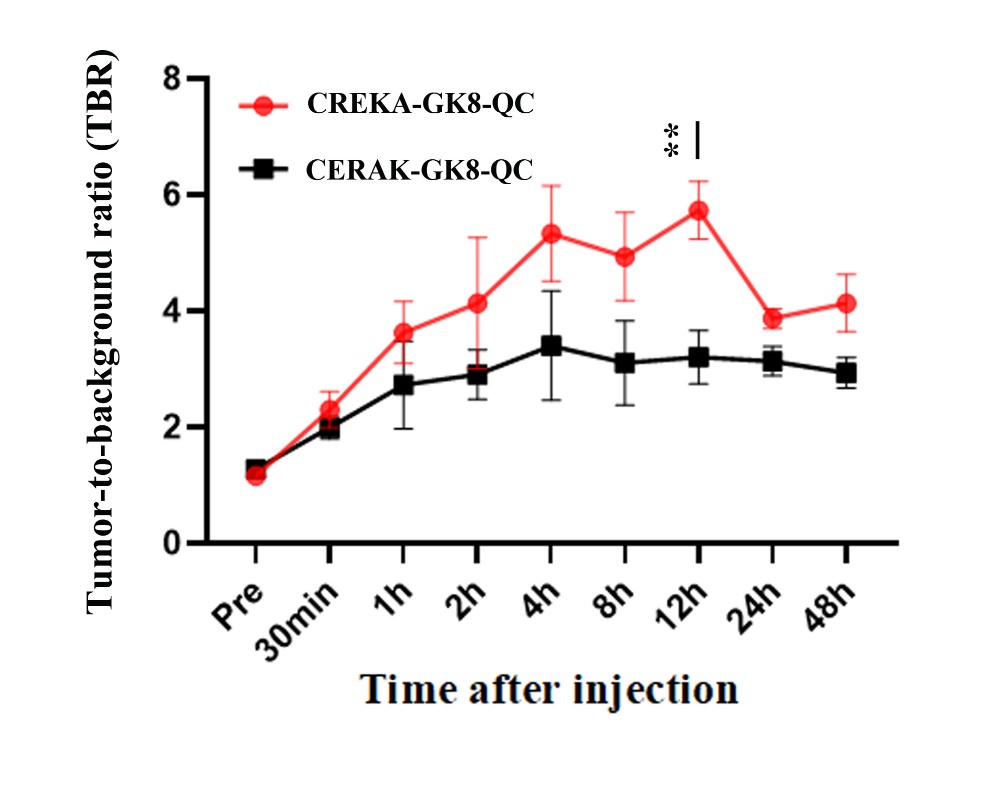


**Fig. S16** *In vivo* TBR at different time points. Quantification of *in vivo* fluorescence imaging TBR. Data represented as the mean ± SD, n=3, **p<0.01.


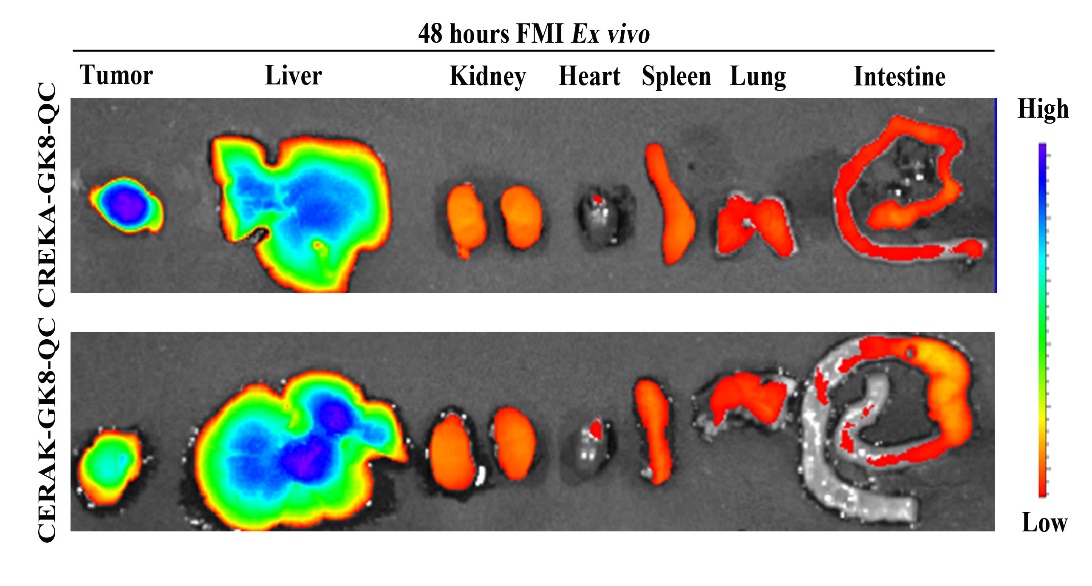


**Fig. S17** *Ex vivo* fluorescence imaging of subcutaneous 4T1 breast tumors and major organs. *Ex vivo* fluorescence imaging of tumors and major organs, including liver, kidney, heart, spleen, lung, and intestine, at 48 hours post-injection of CREKA-GK8-QC or CERAK-GK8-QC.


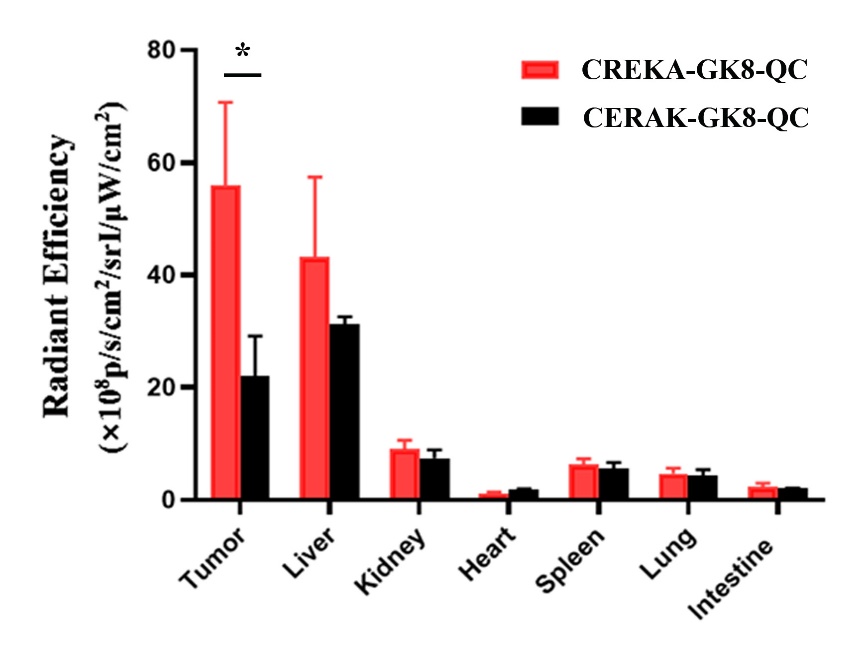


**Fig. S18** Fluorescence intensities of subcutaneous 4T1 breast tumors and major organs. *Ex vivo* fluorescence intensities of tumors and major organs at 48 hours post-injection of CREKA-GK8-QC or CERAK-GK8-QC. Data represented as the mean ± SD, n=3, *p<0.05.


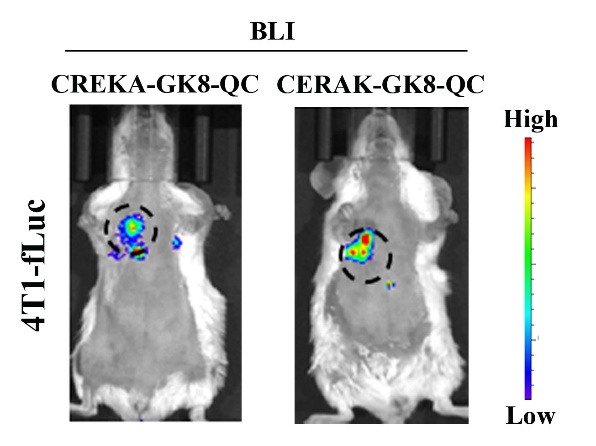


**Fig. S19** BLI imaging of metastatic lung lesions. BLI of lung metastasis in 4T1 tumor bearing mice which was applied to detect the location of metastatic lesions.


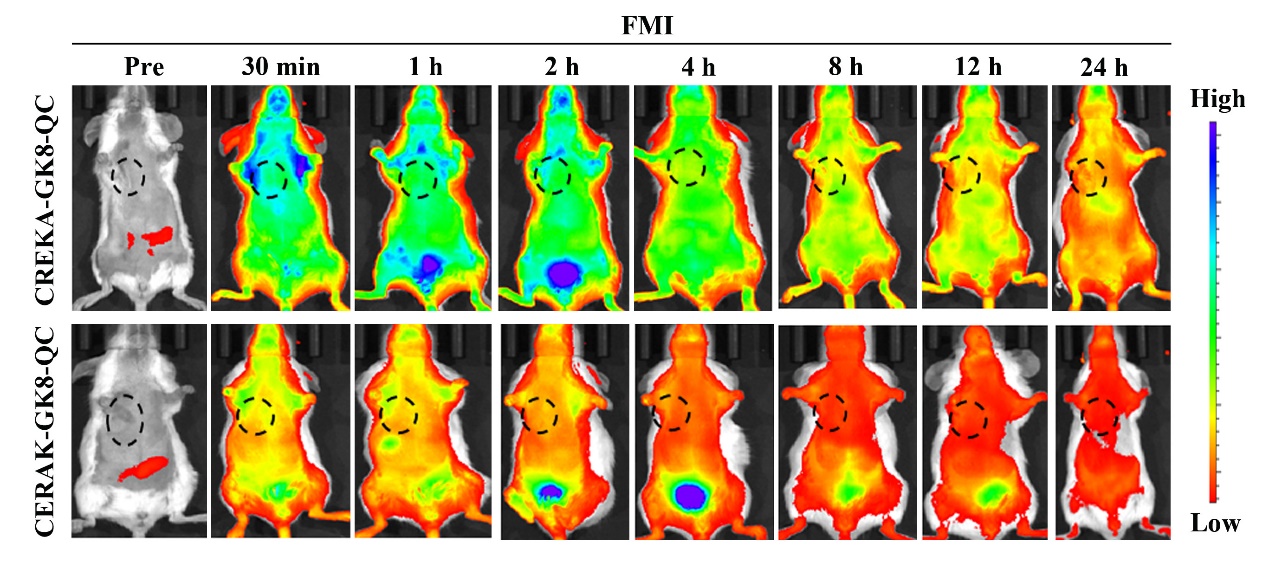


**Fig. S20** *In vivo* fluorescence imaging of metastatic lung lesions**.** *In vivo* fluorescence images of metastatic breast cancer bearing mice injected with CREKA-GK8-QC or CERAK-GK8-QC (n=3) at different time points. The dotted black circles indicated the locations of metastatic lesions.


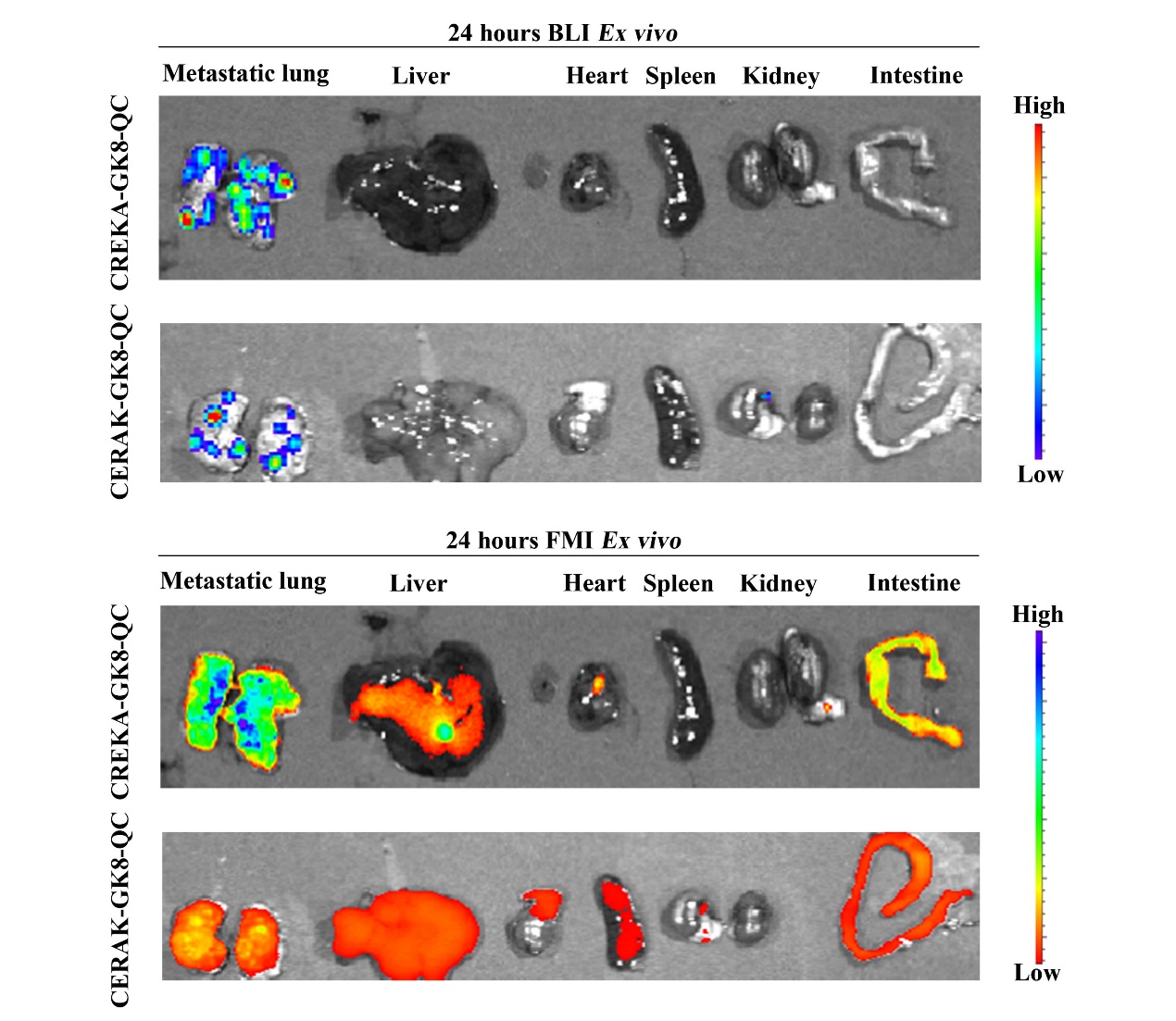


**Fig. S21** *Ex vivo* BLI and fluorescence images of metastatic lung lesions 4T1 tumor bearing mice at 24 h post injection of CREKA-GK8-QC or CERAK-GK8-QC.


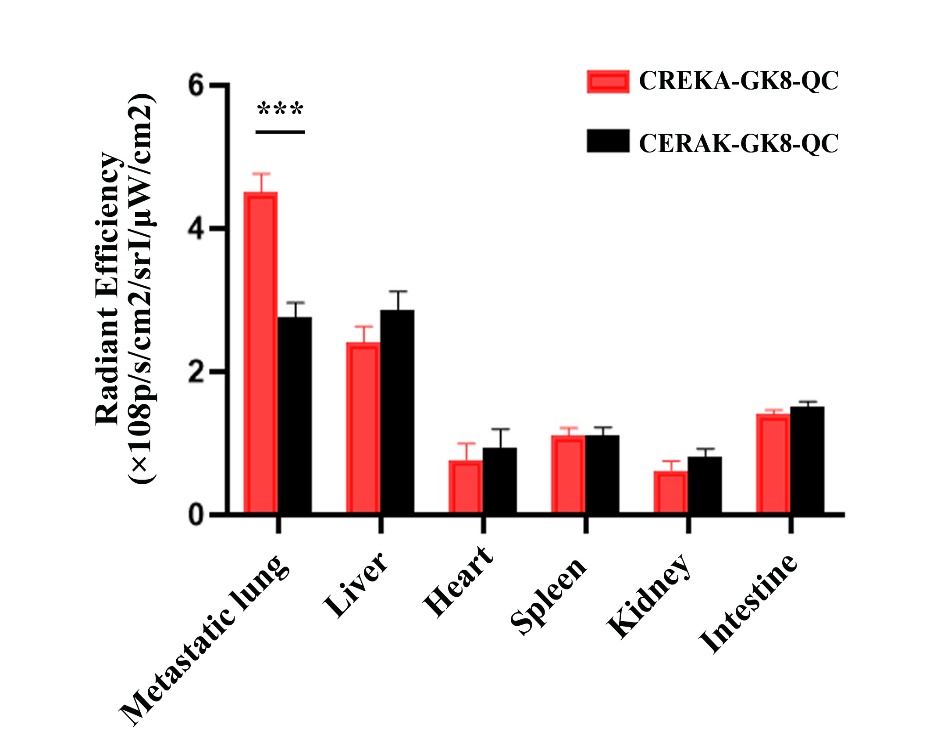


**Fig. S22** *Ex vivo* fluorescence intensities of metastatic lungs and major organs at 24 hours post-injection of CREKA-GK8-QC or CERAK-GK8-QC. Data represented as the mean ± SD, n=3, ***p<0.001.


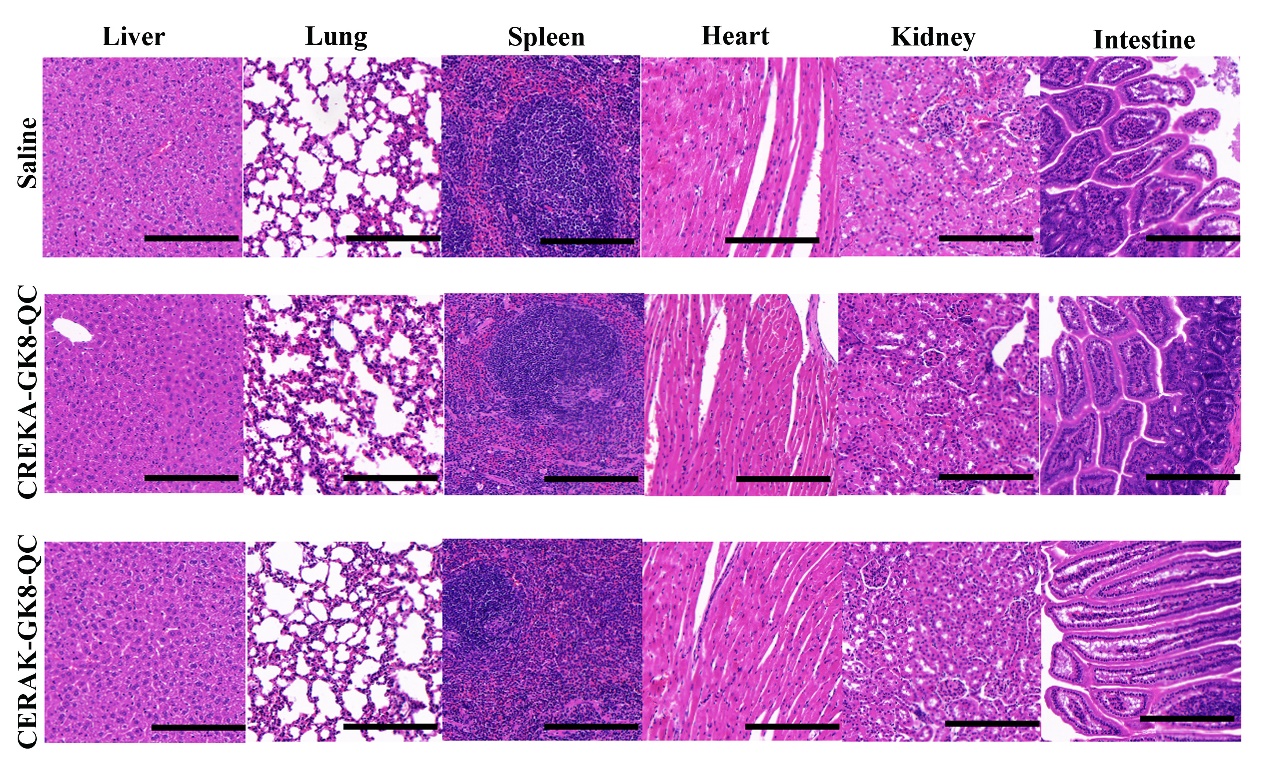


**Fig. S23** Histologic analysis of major organs. HE staining images of major organs (heart, liver, spleen, lung, kidney, and spleen) from different groups. Scale bars: 50 μm.


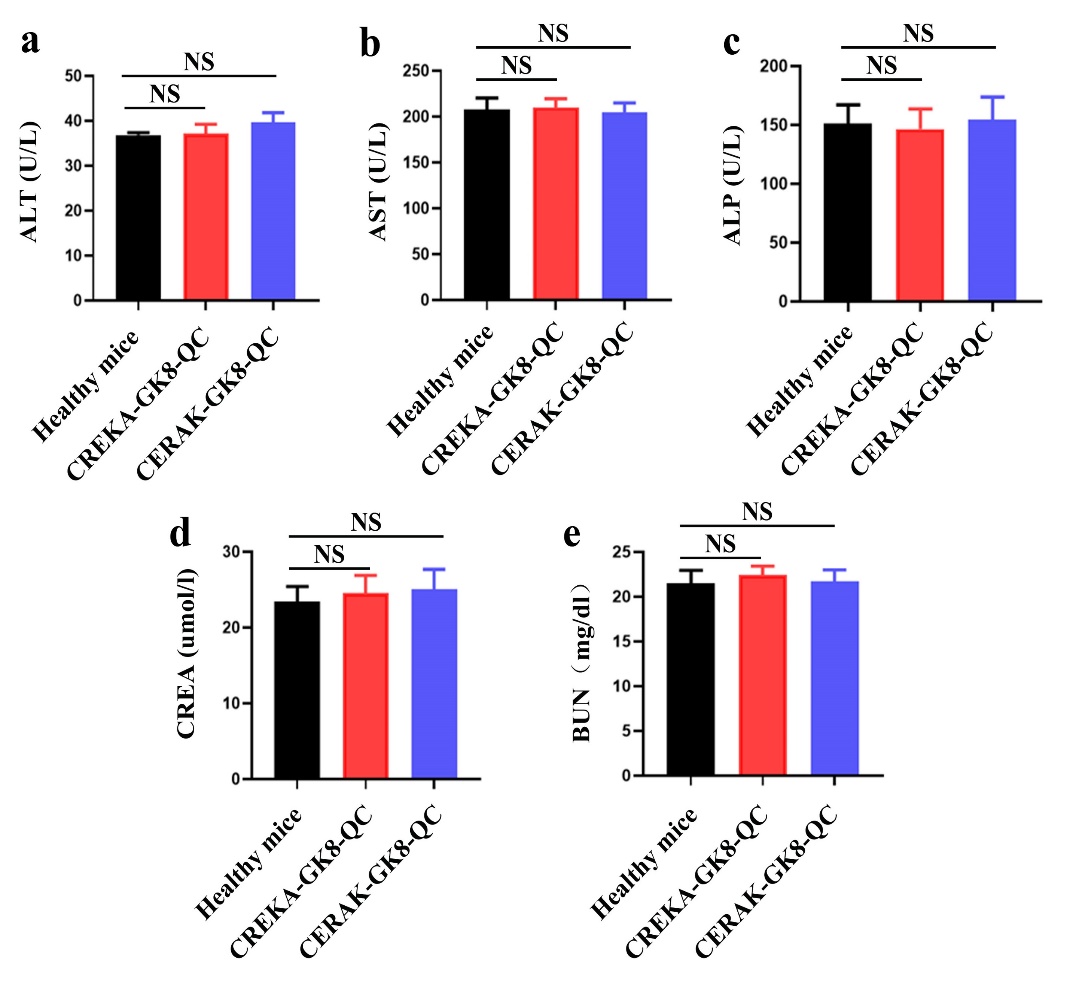


**Fig. S24** Liver and kidney function analysis. Liver function, including **a.** ALT, **b.** AST, and **c.** ALP and kidney function, including **d.** Scr and **e.** BUN, were all analyzed from different groups.


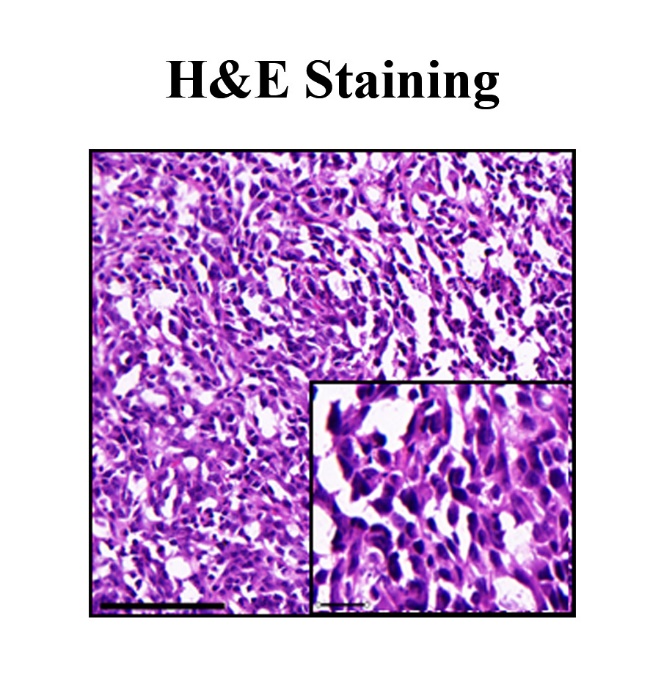


**Fig. S25** H&E staining was utilized to confirm the tumor residuals. Scale bar 50 µm. Inset: enlarged images of the metastasis sections (scale bar 10 µm).

**Table. S1** Local tumor recurrence after surgery

| **Mice** | **Cancer Recurrence** | **Survival Time post-surgery** | **Overall recurrence rate (%)** |
| --- | --- | --- | --- |
| Fluorescence-1 | √ | 22 | 20 |
| Fluorescence-2 | × | >30 |  |
| Fluorescence-3 | × | >30 |  |
| Fluorescence-4 | × | >30 |  |
| Fluorescence-5 | × | >30 |  |
| White light-1 | √ | 21 | 100 |
| White light-2 | √ | 21 |  |
| White light-3 | √ | 23 |  |
| White light-4 | √ | 24 |  |
| White light-5 | √ | 28 |  |

All mice treated with fluorescence-guided surgery and conventional white light surgery were monitored on their cancer recurrence (n=5).
